# Solving the maximum cut problem using Harris Hawk Optimization algorithm

**DOI:** 10.1371/journal.pone.0315842

**Published:** 2024-12-30

**Authors:** Md. Rafiqul Islam, Md. Shahidul Islam, Pritam Khan Boni, Aldrin Saurov Sarker, Md. Asif Anam

**Affiliations:** 1 Department of Computer Science, American International University - Bangladesh, Dhaka, Bangladesh; 2 Department of Computer Science and Engineering, University of Asia Pacific, Dhaka, Bangladesh; 3 Computer Science and Engineering Discipline, Khulna University, Khulna, Bangladesh; Nottingham Trent University School of Science and Technology, UNITED KINGDOM OF GREAT BRITAIN AND NORTHERN IRELAND

## Abstract

The objective of the max-cut problem is to cut any graph in such a way that the total weight of the edges that are cut off is maximum in both subsets of vertices that are divided due to the cut of the edges. Although it is an elementary graph partitioning problem, it is one of the most challenging combinatorial optimization-based problems, and tons of application areas make this problem highly admissible. Due to its admissibility, the problem is solved using the Harris Hawk Optimization algorithm (HHO). Though HHO effectively solved some engineering optimization problems, is sensitive to parameter settings and may converge slowly, potentially getting trapped in local optima. Thus, HHO and some additional operators are used to solve the max-cut problem. Crossover and refinement operators are used to modify the fitness of the hawk in such a way that they can provide precise results. A mutation mechanism along with an adjustment operator has improvised the outcome obtained from the updated hawk. To accept the potential result, the acceptance criterion has been used, and then the repair operator is applied in the proposed approach. The proposed system provided comparatively better outcomes on the G-set dataset than other state-of-the-art algorithms. It obtained 533 cuts more than the discrete cuckoo search algorithm in 9 instances, 1036 cuts more than PSO-EDA in 14 instances, and 1021 cuts more than TSHEA in 9 instances. But for four instances, the cuts are lower than PSO-EDA and TSHEA. Besides, the statistical significance has also been tested using the Wilcoxon signed rank test to provide proof of the superior performance of the proposed method. In terms of solution quality, MC-HHO can produce outcomes that are quite competitive when compared to other related state-of-the-art algorithms.

## Introduction

The Max-Cut problem is a famous combinatorial optimization challenge in graph theory. It entails partitioning the vertices of an undirected graph into two separate sets, to maximize the number of edges that cross between these sets. In simpler terms, the goal is to split the graph’s vertices into sets A and B in a way that maximizes the number of edges connecting vertices from different sets. This problem is formally expressed as an optimization task, where the objective is to find the partition that yields the maximum number of cut edges. It’s worth noting that the Max-Cut problem is one of the known NP-hard problems, signifying its computational complexity. [1].

The max-cut problem is remarkable for modeling other combinatorial issues and real-world applications. Many researchers have shown their interest in the Max-cut problem due to its complexity and applications. The Max-cut problem plays an important role in graph theoretic applications. In addition to its theoretical significance, the Max-Cut problem finds practical utility across a range of domains, including but not limited to network design, statistical physics [2], VLSI design [3], circuit layout design, and the production of printed circuit boards [3]. This problem has been instrumental in addressing challenges like data clustering [4], numerical computations, scientific computing, and the hybridization of techniques such as the cross-entropy method [5].

Many techniques were developed to solve the Max-cut problem. Many algorithms were proposed by the researchers, for example, GA (Genetic Algorithm) [6], SA (Simulated Annealing) [7], scatter search [8], Tabu Search [9], rank-two relaxation heuristic [10] filled function method [11], Weakly Bipartite Graphs [12], Path-Relinking (PR) intensification [13]. All of the proposed algorithms have played a great role in their places to solve the Max-cut problem. Although many algorithms were proposed by the researchers to solve the Max-cut problem, none of them were able to provide the best-known results for all the instances of the benchmark datasets.

A well-defined meta-heuristic method can be used to solve the maximum cut problem. As a result, we are trying to solve the problem by applying the Harris Hawk Optimization (HHO) algorithm to get better results. Because it has a particular searching capacity, the HHO method is more efficient than other meta-heuristic approaches in terms of reducing execution time without losing prediction accuracy [1]. Many optimization problems were solved by HHO in recent years, such as image segmentation [14], numerical and engineering optimization [15], SVM for drug design and discovery [16], information exchange [17], parameter estimation of photovoltaic models [18], simulated annealing for feature selection in the medical field [19], Gaussian Mutation [20], Parameters extraction of three-diode photovoltaic model [21], hyperparameters optimization to detect Covid 19 from chest images [22], etc. with better results than the other existing meta-heuristics algorithms. The supremacy of HHO is also encountered by hybridizing with the enhanced Chimp algorithm for protecting copyright in color images [23]. Besides, the amalgamation of two or more metaheuristic algorithms can provide better outcomes that can be proved by many contemporary research works. Such as, the whale optimization algorithm is hybridized with the salp swarm algorithm to provide better optimization outcomes in Improved Whale Optimization Salp Swarm algorithm (IWOSSA) [24]; grid search and Aquila Optimizer (AO) are bounded to obtain optimized hyperparameters of ML and CNN models to recognize heart diseases [25]; the amalgam of genetic algorithm and particle swarm optimization has been used for tuning the adaptive PI controller [26]. Thus, in the current research work, two operators of the chemical reaction optimization algorithm (CRO) are used along with the operators of HHO to obtain better solutions. The contributions of the work are as follows.

1. To generate the initial population we modified the graph as a hawk and produced a suitable solution space for solving the Max-cut problem using HHO.
2. The operators of HHO are redesigned in this paper to obtain the optimized solution from the initial population.
3. HHO is hybridized with two of the operators of the chemical reaction optimization (CRO) algorithm which are refinement and crossover. These two operators help to find better solutions by searching globally from the search space.
4. To enhance the partition of the graph, the Kernighan-Lin graph partitioning algorithm is modified in this problem.
5. The performance of the HHO algorithm is intensified by using a repair operator that searches locally from the solution space.
6. The experimental result of the proposed approach is compared with TSHEA and PSO-EDA.
7. Wilcoxon signed ranked test is provided for supporting the supremacy of the experimental result of the proposed method.

### Problem statement and objective function

The Max-Cut problem is not only a fundamental graph partitioning challenge but also one of the most formidable combinatorial optimization problems to tackle. Its primary objective is to partition the vertex set of a graph into two subsets in a way that maximizes the total weight of edges with one endpoint in each subset. The Max-Cut problem is classified as NP-complete, meaning it is both a part of the NP complexity class, and it’s challenging to find an optimal solution.

Let, there be a graph, *G* = (*V*, *E*), where *V* is the set of vertices {1, 2, …, *n*} and *E* is the set of edges where each edge (*i*, *j*) ∈ *E* is associated with a weight *W*_*ij*_, the problem aims to divide the graph G into two partitions, *S* and *V*\*S*, such that the sum of edge weights in the cut is maximized.

For a graph with nodes, *v*_1_, *v*_2_, *v*_3_, *v*_4_, and *v*_5_, the max cut algorithm cuts the edges of the graph in such a way that the cut is maximum, i.e., the sum of the weighted edges that have been cut is maximum.

**Fig 1:**
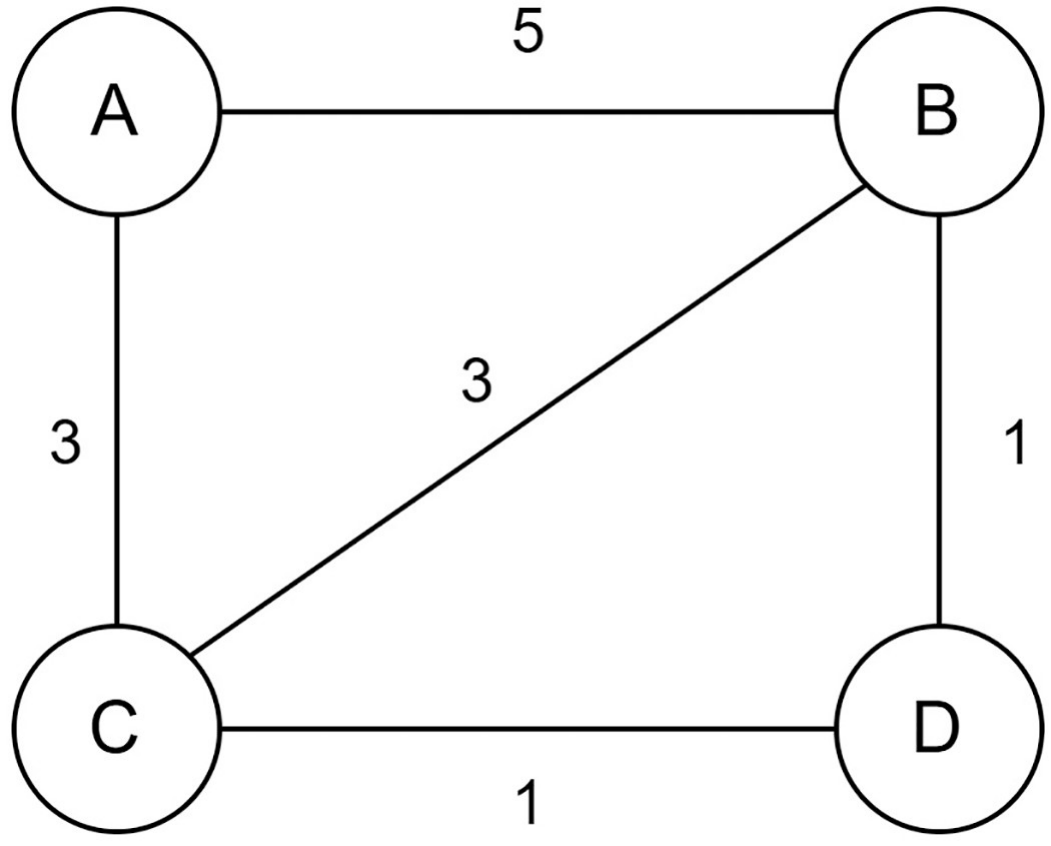
An example graph.

In Fig 1 a graph has been shown where the graph is denoted as *G* = (*V*, *E*), *V* = {*A*, *B*, *C*, *D*} and *E* = {(*AB*, 5), (*AC*, 3), (*BC*, 3), (*BD*, 1), (*CD*, 1)}. The graph can be cut in many ways. All of the cuts are shown in Table 1.

**Table 1:** Feasible solutions for the graph of Fig 1.

Subset, *S*	Subset,*V*\*S*	Cut edges	Cut edges value
{A}	{B, C, D}	{AB, AC}	8
{B}	{A, C, D}	{AB, BC, BD}	9
{C}	{A, B, D}	{AC, BC, CD}	7
{D}	{A, B, C}	{BD, CD}	2
{A, B}	{C, D}	{AC, BC, BD}	7
{A, C}	{B, D}	{AB, BC, CD}	9
**{A, D}**	**{B, C}**	**{AB, AC, CD, BD}**	**10**

**Fig 2:**
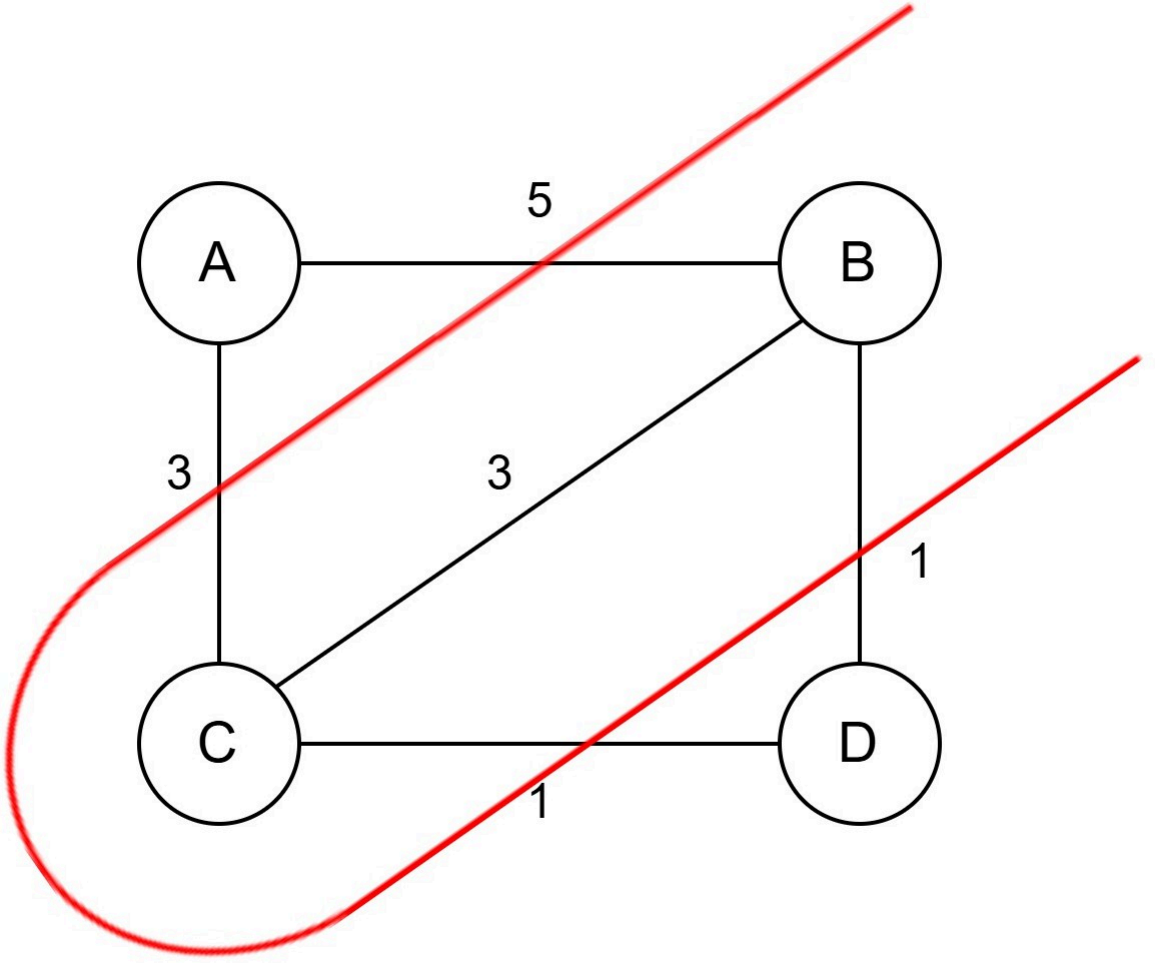
A graph with maximum cut.

According to Table 1 the maximum cut of the graph of Fig 1 is 10. The best solution is found by cutting down the maximum weight that is shown in Fig 2. Here, the maximum cut of the edges = AB, AC, CD, BD, and the sum of the cut edges is 5 + 3 + 1 + 1 = 10, which is the maximum.

Given a graph, *G* = (*V*, *E*), where *V* is the set of vertices {1, 2, …, *n*} and *E* is the set of edges where each edge (*i*, *j*) ∈ *E* is associated with a weight *W*_*ij*_. The problem aims to divide graph *G* into two partitions, *S* and *V*\*S*, such that the sum of edge weights in the cut is maximized. After the cut, each edge connecting i and j should be divided such that some parts will belong to the subset *S* while the remaining parts of the edge should be in the subset *V*\*S* in *E*. The set of edges in the cut graph is *E*′ = *e*_*i*,*j*∈*E*,*i*∈*S*,*j*∈*V*\*S*_. The objective function of the max cut problem is as follows as given in [27].

$$\begin{array}{c} f\left(x\right)=max\sum_{i\in S,j\in V\backslash S}W_{ij} \end{array}$$ (1)

## Related work

The Max-Cut problem has been the subject of various techniques and algorithms aimed at optimization. Each approach has its own merit and has found its niche. Every algorithm comes with its set of strengths as well as weaknesses or limitations.

Svatopluk Poljak and Franz Rendl has solved the Max-Cut problem using eigenvalue relaxation [14]. Their objectives have two main aspects—efficiently computing the constraint and enhancing its quality. The authors provide a comprehensive account of the method, covering its theoretical underpinnings, algorithmic implementation, and operational efficacy. They also leveraged the fundamental algorithm to calculate both upper and lower bounds on the max-cut, noting that, in most cases, the relative disparity between these bounds was notably less than 10%. To obtain precise max-cut values, the authors also applied the fundamental algorithm within a “branch and bound” context. They successfully solved the max-cut problem for dense geometric networks with up to 100 nodes and conducted a comparative analysis with the Kernighan-Lin local search algorithm. The eigenvalue bound introduced here is established as a potent tool for addressing max-cut problems. It is worth noting that the experiments detailed in the presented paper do not push the eigenvalue bound to its limits. The polyhedral approach excels particularly in extremely sparse graphs, as the computational effort for the LP relaxation is independent of the vertex count (|*V*|) but does heavily depend on the edge count (|*E*|).

In 2012, Q. Wu et al. introduced a hybrid evolutionary algorithm (TSHEA) based on tabu search to address the Max-Cut problem, aiming to reduce edge count bias and improve the results [15]. TSHEA employs a combination operator, merging a one-flip and confined exchange move neighborhood with a distance-and-quality-based solution approach. TSHEA distinguishes itself in several ways: it utilizes neighborhood combination in its tabu search procedure, employs a solution combination operator similar to traditional uniform crossover using two parent solutions, and performs well on larger benchmark instances, consistently matching or surpassing the best-known solutions. The approach outperforms other reference algorithms from the literature, discovering new optimal solutions in numerous instances. However, it may require a high number of iterations and has several tunable parameters.

Lin Geng et al. [17] introduced PSO-EDA, a hybrid approach for the max-cut problem that combines particle swarm optimization and estimate of distribution algorithm. It outperforms previous methods in most cases but faces challenges in high-dimensional spaces.

Sú-Hyang Kim et al. [20] proposed a hybrid genetic algorithm for max-cut graph partitioning, showing significant improvements over existing algorithms. They incorporated unique ratio gain measures for vertex movement. The algorithm works perfectly on the sparse graphs but fails to produce good results in the dense graphs.

Sahni and Gonzalez [28] developed a randomized greedy algorithm for the max-cut problem. Goemans and Williamson [29] improved its performance by using a more complex distribution, achieving a 0.878 approximation. They represented vertices as unit vectors and employed semidefinite programming, pioneering its use in approximation algorithms. This work tackled fundamental questions in approximation algorithms.

For locating approximations of solutions to this optimization issue, the authors presented a heuristic technique based on the scatter-search approach [30]. Within the scatter-search framework, their solution method included a few cutting-edge elements. To improve variety in the reference set, three things must happen that are (1) the maximum diversity problem must be solved; (2) a key search parameter must be dynamically adjusted; and (3) a combination method must be adaptively chosen. They carried out extensive computational tests to evaluate the effectiveness of their proposal with earlier solution techniques after first examining the impact of changes in crucial scatter-search aspects. The construction of a scatter-search (SS) approach that incorporates a few novel components could lead to the conventional methodology serving as the inspiration for their work. Providing a method for the max-cut problem that could generate accurate approximations in a reasonable period of computer time was also a key objective. Three distinct techniques for extending the fundamental scatter search implementation were used in this study. A novel selection method for creating a reference set out of a population of solutions made up of the initial extension. The depth parameter k related to the ejection chain mechanism was dynamically adjusted in their second extension. In the third extension, the combination strategies were chosen probabilistically. The likelihood of choosing any one of the three approaches was inversely correlated with the number of excellent solutions those approaches had produced in earlier iterations. Performance was more developed than in previous works. The results that we obtained with their SS implementation were not all due to the strategies that they tested.

In this study, the authors explored the Max-cut problem capabilities of the ant colony optimization (ACO) heuristic and provided an Ant-Cut algorithm [31]. AntCut employs the ACO heuristic to select a vertex to change the set that it belongs to at each step as it moves closer to the maximum cut. By altering the set that the chosen vertex is a part of, the likelihood of each vertex should be picked in a manner that is proportionate to the cut’s rising weight. They conducted tests using the graphs G1, and G11 in the G-set; Helmberg and Rendl created the test issues for the G-set using a Rinaldi-written graph generator. The majority of max-cut problems can be successfully solved using the AntCut algorithm. The performance of the ACO algorithm may decrease over time.

Besides, in recent times, the Max-cut problem has been solved using supervised and reinforcement learning [32], in which the authors suggested a hybrid approach that utilized the pointer network with the supervised and reinforcement learning techniques. On the other hand, Hassin et al. [33] proposed a solution to this problem that incorporates the greedy heuristic approach with edge-contraction heuristic with differencing method. However, they were not able to find the reason behind the better performance of the method properly. Quantum approximate optimization algorithm (QAOA) is also used to solve the problem and Bae et al. [34] provide the proof that on complete graphs recursive QAOA performs better than original QAOA. Although they provide the proof analytically for complete graphs there is no proof regarding sparse graphs for real-time datasets.

While various algorithms have been developed to address the Max-Cut problem, there are still several areas for improvement. Many algorithms, such as eigenvalue relaxation, scatter-search, and hybrid evolutionary algorithms like TSHEA and PSO-EDA, have shown promising results. However, most of these algorithms are applied only to smaller datasets and face significant challenges in finding the Max-Cut for dense graphs. Additionally, evolutionary algorithms typically rely on extensive parameter tuning. Therefore, the research gap lies in developing a robust and scalable algorithm that performs efficiently across both dense and sparse graph instances and minimizes computational complexity, without a heavy reliance on parameter tuning.

Meta-heuristics have gained popularity for solving complex optimization problems due to their ability to navigate large and often irregular solution spaces efficiently. These algorithms are designed inspired by natural processes, including biological evolution, animal behavior, and physical systems. In past decades, numerous meta-heuristics have been developed, each with its unique approach to balancing exploration and exploitation. In Table 2, we present a comparative analysis of some widely used meta-heuristics, highlighting their key strengths, weaknesses, and typical applications.

**Table 2:** Comparison of popular meta-heuristics.

Meta-Heuristic	Year of Publication	Strengths	Weaknesses
**Genetic Algorithm (GA)** [35]	1975	Good for global optimization, flexible, easy to parallelize	Slow convergence, sensitive to parameter settings
**Particle Swarm Optimization (PSO)** [18]	1995	Simple framework, few parameters, fast convergence	Prone to local optima, requires fine-tuning of parameters
**Simulated Annealing (SA)** [36]	1983	Avoids local optima, versatile across various problem domains	Slow convergence, requires careful cooling schedule
**Ant Colony Optimization (ACO)** [37]	1992	Good for discrete optimization, exploits previous solutions effectively	Slow convergence, complex parameter tuning
**Harris Hawk Optimization (HHO)** [38]	2019	Strong exploration and exploitation balance, simple to implement	Sensitive to initial population, may converge prematurely
**Chemical Reaction Optimization (CRO)** [39]	2010	Flexible, powerful operators, adaptable to various problem domains	High computational cost, sensitive to reaction parameters
**Firefly Algorithm (FA)** [40]	2008	Good for multimodal optimization, easy to adapt	Prone to premature convergence, sensitive to light intensity parameters
**Tabu Search (TS)** [41]	1986	Explores local neighborhoods efficiently, avoids cycling through solutions	Dependent on tabu list length, can get stuck in local optima
**Cuckoo Search (CS)** [42]	2009	Fast convergence, efficient in avoiding local optima	Poor diversity in solutions, limited application to constrained problems
**Artificial Bee Colony (ABC)** [43]	2005	Efficient for multi-objective problems, good balance between exploration/exploitation	Requires large population size, sensitive to initial solutions
**Grey Wolf Optimizer (GWO)** [44]	2014	Simple, mimics natural behavior, fewer parameters to adjust	Sensitive to initial population, can converge prematurely
**Bat Algorithm (BA)** [45]	2010	Good convergence speed, balances exploration/exploitation well	Parameter sensitivity, relies on good parameter settings
**Whale Optimization Algorithm (WOA)** [46]	2016	Good for multimodal and scalable problems	Converges slowly for large-scale problems

## Harris Hawk Optimization (HHO) for the max-cut problem

While numerous algorithms have been developed to address the max-cut problem, not all of them consistently yield the best solutions across all dataset instances. In this section, we present our proposed approach for solving the maximum cut problem. We employ the Harris Hawk Optimization (HHO) algorithm, which is detailed in the following subsections.

### Harris Hawk Optimization

In 2019, Ali Asghar Heidari et. al. proposed the Harris Hawk Optimization (HHO) algorithm which has been used to solve many engineering problems ever since [38]. The HHO is a metaheuristic technique that excels in terms of search performance. Its four exploitation methods may search solution space both locally and globally which makes the algorithm more efficient than any other one.

The HHO algorithm is inspired by the intelligent hunting behavior of the Harris hawk which hunts its prey in a team. The prey is normally small animals like a rabbit. The hawks explore desert sites for hours, and when they detect the prey, they encircle the prey and exploit it until the prey has low energy so the hawks can easily catch it.

There are three phases in the HHO algorithm. They are:

- **Exploration phase**: This is the phase where a hawk explores and searches for a potential target. The hawks can wait several hours before prey is finally found. When a rabbit is detected, the algorithm enters the exploitation phase.
- **Transition phase**: This phase is intermediate between exploration and exploitation. When a hawk detects prey, it alerts the other hawks and starts hunting.
- **Exploitation phase**: This is when the group of hawks uses strategies to kill the prey depending on the fitness value of the hawk and the escaping energy of the prey. The hawks chase the rabbit from different directions for a long time, which reduces the rabbit’s energy. In the end, the hawk cannot run anymore as it is tired, and then the hawks strike the killing bow.

### Basic structure of the max-cut Problem using HHO algorithm

The main method we have applied for finding the maximum cut problem is the Harris Hawk Optimization (HHO) algorithm. First of all, we have generated an initial population. After that, the hawks enter into the HHO phases—the exploration, transition phase, and exploitation phases. After these phases, HHO operators are applied to it to improve the result.

Some of the parameters used in the Harris Hawks Optimization (HHO) algorithm are described in Table 3.

**Table 3:** Some initial parameters of HHO.

Parameter	Description
*X*_*i*_	Location of *i*^*th*^ hawk
*X*_*i*(*t*+1)_	Updated position of *i*^*th*^ hawk in the next iteration
*X*_*rand*_	Location of a random hawk
*X*_*m*(*t*)_	Average position of hawks
*Sizepop*, *N*	Total number of populations
*r*_1_, *r*_2_, *r*_3_, *r*_4_, *q*	Random values within the range [0, 1]
*UB*	Upper bound
*LB*	Lower bound

### Initial population generation

The initial population represents the initial positions of the hawks. It is generated based on random selection. At first, we have taken the input *N*, the total number of hawks. We then create random hawks by randomly removing edges to generate a random graph cut. In the HHO phase, the cut is improved in each iteration. A variable *T* limits the maximum number of iterations. In each iteration, the positions of the hawks are updated. The initial population generating process is shown in Algorithm 1.

**Algorithm 1**: Initial population generation.

1 **Input**: Graph, G = (V, E)

2 **Output**: modified graph as a hawk

3 **Init**: A, B

4 **for** *each v* ∈ *V* **do**

5  r ← random([0, 1])

6  **if** *r* ≥ 0.5 **then**

7   *A* ← *v*

8  **end**

9  **else**

10   *B* ← *v*

11  **end**

12 **end**

13 **for** *x* ∈ *A* **do**

14  **for** *y* ∈ *B* **do**

15   Assign weight of edge E(x, y) ← 0

16  **end**

17 **end**

### HHO phases

The phases of HHO are discussed below with necessary diagrams and pseudocodes.

#### Phase 1: Exploration phase

This phase describes how a Harris hawk explores the search space for prey. It includes positioning the hawks randomly and waiting for several hours. Here, hawks are agents, and the position of the prey is the best candidate solution. Detecting prey depends on two strategies. The first strategy (when *q* < 0.5) specifies detecting prey according to the positions of other hawks (*X*_*i*_, *i* = 1, 2, 3, …, *N* where *N* is the total number of Hawks). The second strategy (when *q* ≥ 0.5) specifies the detection of prey according to perching on a random tree *X*_*rand*_. These two strategies are modeled in the Eq 2.

$$\begin{array}{c} X_{i}\left(t+1\right)=\{\begin{array}{cc} X_{rand}\left(t\right)-r_{1}\left|X_{rand}\left(t\right)-2r_{2}X\left(t\right)\right| & \text{if}\;q\geq 0.5\\ X_{rabbit}\left(t\right)-X_{m}\left(t\right)-r_{3}\left(LB+r_{4}\left(UB-LB\right)\right) & \text{if}\;\text{q}\;<\;\text{0.5} \end{array} \end{array}$$ (2)

*X*_*m*_ is calculated as follows.

$$\begin{array}{c} X_{m}\left(y\right)=\frac{\sum_{i=1}^{N}X_{i}\left(t\right)}{N} \end{array}$$ (3)

The description of the variables used in the Exploitation phase is provided in Table 4.

**Table 4:** Description of variables used in the exploitation phase.

Variables	Description	Values
*X*_*rand*_	Location of random hawk	Randomly generated
*X*(*t*)	Current position vector of hawk	Randomly generated
*X*_*i*_	Location of ith hawk	Randomly generated
*X*_*rabbit*_	Location of the prey	Randomly generated
*X*_*m*_	Average position of hawk	From Eq 2
*LB*	Lower bound	Selected
*UB*	Upper bound	Selected
*r*_1_, *r*_2_, *r*_3_, *r*_4_, *q*	Random values	Random values within 0 to 1

#### Phase 2: Transition phase

This is an intermediate phase between the exploration phase and the exploitation phase. This transition phase is formulated as follows:

$$\begin{array}{c} E=2E_{0}{\left(1-T\right)}^{t} \end{array}$$ (4)

Here *E* is the escaping energy of the prey, *E*_0_ is the initial energy, *t* is the current number of iterations and *T* is the maximum iteration. The value of *E*_0_ varies in the range from [-1, 1]. When the value increases from 0 to 1, the prey is strengthening, and in the case of decreasing value from 0 to –1, the prey is flagging. When |*E*| ≥ 1, the algorithm enters the exploration phase, otherwise, it moves to the exploitation phase. The description of the variables used in the Transition phase is provided in Table 5.

**Table 5:** Description of variables used in transition phase.

Variables	Description	Values
E	Current energy of the prey	Calculated from Eq 3
E0	Initial energy of the prey	Random number from -1 to 1
T	Maximum number of iterations	Selected
t	Current iteration	Increment in each loop iteration

#### Phase 3: Exploitation phase

The chasing strategies of the hawks and the escaping behaviors of the prey are the two main elements of this phase. There are four attack strategies of the hawks depending on the energy of the prey.

- **Strategy 1: (Soft Besiege)** When the escaping energy of the prey is enough and tries to escape by some random jumps, but in the end cannot escape successfully, that is |*E*| ≥ 0.5 and *r* ≥ 0.5. The hawks can easily hunt prey down. The equations are:
  $$\begin{array}{c} X_{i}\left(t+1\right)=\Delta X\left(t\right)-E\left|JX_{target}\left(t\right)-X\left(t\right)\right| \end{array}$$ (5)
  $$\begin{array}{c} \Delta X\left(t\right)=X_{rabbit}\left(t\right)-X\left(t\right) \end{array}$$ (6)
  Here, Δ*X*(*t*) represents the difference between the position vector and current location in the iteration t and jump strength of the rabbit during the escaping procedure, *J* = 2(1 − *r*5) where *r*5 is a random variable inside the range [0, 1]. The value of *J* changes randomly in each iteration.
  The description of the variables used in the Soft Besiege is provided in Table 6.
- **Strategy 2: (Hard Besiege)** When the prey is exhausted and cannot escape due to a lower energy, this strategy is used. The current positions are updated using the following equation:
  $$\begin{array}{c} X_{i}\left(t+1\right)=X_{rabbit}\left(t\right)-E\left|\Delta X\left(t\right)\right| \end{array}$$ (7)
  The description of the variables used in the Hard Besiege is provided in Table 7.
- **Strategy 3: (Soft Besiege with Progressive Rapid Dives)** This strategy is used when the prey has enough escaping energy, but the hawks still construct a soft besiege before the surprise pounce. This procedure is more intelligent than the soft besiege.
  $$\begin{array}{c} Y=\Delta X_{rabbit}\left(t\right)-E\left|JX_{rabbit}\left(t\right)-X\left(t\right)\right| \end{array}$$ (8)
  To model the escaping of the prey, the Levy Flight concept is utilized in HHO. The Levy Flight is utilized to mimic random jumping and zigzagging to escape. The prey escapes using this equation:
  $$\begin{array}{c} Z=Y+S\times LF(D) \end{array}$$ (9)
  where *D* is the dimension of the problem, *S* is a random vector of size 1 × *D* and *LF* is the Levy Flight function, which is calculated as follows [47]:
  $$\begin{array}{c} LF\left(X\right)=\frac{\mu }{{\left|\nu \right|}^{1/\beta }} \end{array}$$ (10)
  where *μ* and *ν* are random variables drawn from normal distributions $\mu ∼N\left(0,\sigma_{\mu }^{2}\right),\;\nu ∼N\left(0,\sigma_{\nu }^{2}\right)$, *β* is the Levy distribution parameter in the range 1 < *β* ≤ 2, and
  $$\sigma_{\mu }=(\frac{\Gamma \left(1+\beta \right)\text{sin}(\frac{\pi \beta }{2})}{\Gamma (\frac{1+\beta }{2})\beta 2^{\frac{\beta -1}{2}}}{)}^{\frac{1}{\beta }},\;\sigma_{\nu }=1$$
  The hawks rapidly dive to make the prey tired and catch it. The next move of the hawk can be decided by the following rule:
  $$\begin{array}{c} Y=X_{rabbit}\left(t\right)-E\left|X_{rabbit}\left(t\right)-X\left(t\right)\right| \end{array}$$ (11)
  Hence, the final strategy for updating the Hawks’ position can be performed by:
  $$\begin{array}{c} X\left(t+1\right)=\{\begin{array}{cc} Y & \text{if}\;\text{F}(\text{Y})<\text{F}(\text{X}(\text{t}))\\ Z & \text{if}\;\text{F}(\text{Z})<\text{F}(\text{X}(\text{t})) \end{array} \end{array}$$ (12)
- **Strategy 4: (Hard Besiege with Progressive Rapid Dives)** When escaping energy is less than 50% (|*E*| < 0.5) and the escape probability is also less than 50% (*r* < 0.5), the hawks employ a hard besiege strategy before launching a surprise attack to capture the prey. The dynamics for the prey during this stage are similar to those in the soft besiege condition. However, in a hard besiege, the hawks actively work to reduce the distance between their average location and the escaping prey. To achieve this, they follow a specific rule set during the hard besiege condition.
  $$\begin{array}{c} X\left(t+1\right)=\{\begin{array}{cc} Y & \text{if}\;\text{F}(\text{Y})<\text{F}(\text{X}(\text{t}))\\ Z & \text{if}\;\text{F}(\text{Z})<\text{F}(\text{X}(\text{t})) \end{array} \end{array}$$ (13)
  where,
  $$\begin{array}{c} Y=X_{rabbit}\left(t\right)-E\left|JX_{rabbit}\left(t\right)-X_{m}\left(t\right)\right| \end{array}$$ (14)
  *X*_*m*_(*t*) is obtained using Eq (3) and Z is obtained using Eq (9).
  The description of variables used in Soft Besiege and Hard Besiege with Progressive Rapid Dives phases is provided in Table 8.

**Table 6:** Description of variables used in soft besiege phase.

Variables	Description	Values
*X*_*i*_(*t* + 1)	Location of *i*^*th*^ hawk	Randomly generated
*X*_*rabbit*_	Location of the prey	Randomly generated
*X*(*t*)	Current position vector of hawk	Randomly generated
Δ*X*(*t*)	Difference between the position vector of the rabbit and current location of the hawk	Calculated from Eq 5

**Table 7:** Description of variables used in hard besiege phase.

Variables	Description	Values
*X*_*i*_(*t* + 1)	Location of *i*^*th*^ hawk	Randomly generated
*X*_*rabbit*_	Location of the prey	Randomly generated
*E*	Current energy of the prey	Calculated from Eq 3
Δ*X*(*t*)	Difference between the position vector of the rabbit and current location of the hawk	Calculated from Eq 5

**Table 8:** Description of variables used in soft besiege and hard besiege with progressive rapid dives phases.

Variable	Description	Value
*X*_*i*_(*t* + 1)	Location of *i*^*th*^ hawk	Randomly generated
*X*_*rabbit*_	Location of the prey	Randomly generated
*X*_*m*_	Average position of hawk	From Eq 2
*E*	Current energy of the prey	Calculated from Eq 3
*X*(*t*)	Current position vector of hawk	Randomly generated
*Z*	Move of hawk	Calculated from Eq 8
*Y*	Move of hawk	Calculated from Eq 7
*D*	Dimension	Selected
*S*	Random vector	Random vector by Size 1 × *D*
*LF*(*D*)	Levy flight function	Calculated from Eq 9
*u*, *v*	Random values	Range within (0, 1)
*β*	Constant value	1.5
Δ*X*(*t*)	Difference between the position vector of the rabbit and current location of the hawk	Calculated from Eq 5

### Modified Kernighan-Lin graph partitioning algorithm

The algorithm takes an undirected graph *G* = (*V*, *E*) with vertex set *V*, edge set *E*, and optional edge weights as input. Its objective is to minimize the sum *T* of the weights of the subset of edges that cross from one partition, *A*, to another, *B*, while dividing the vertex set *V* into two disjoint subsets, *A* and *B*, of approximately equal size. If the graph has no edge weights, the goal is to minimize the number of crossing edges, which is equivalent to assigning each edge a weight of 1. The algorithm continuously enhances the partition by using a greedy algorithm in each iteration to match vertices from *A* with vertices from *B* in a way that shifting the paired vertices between the partitions improves the partition quality. It then selects a subset of pairs that have the most favorable impact on the overall solution quality *T* after vertex matching. Each pass of the procedure takes place in $O(v^{2}\;\text{log}\;v)$ time for a network with *v* vertices.

**Fig 3:**
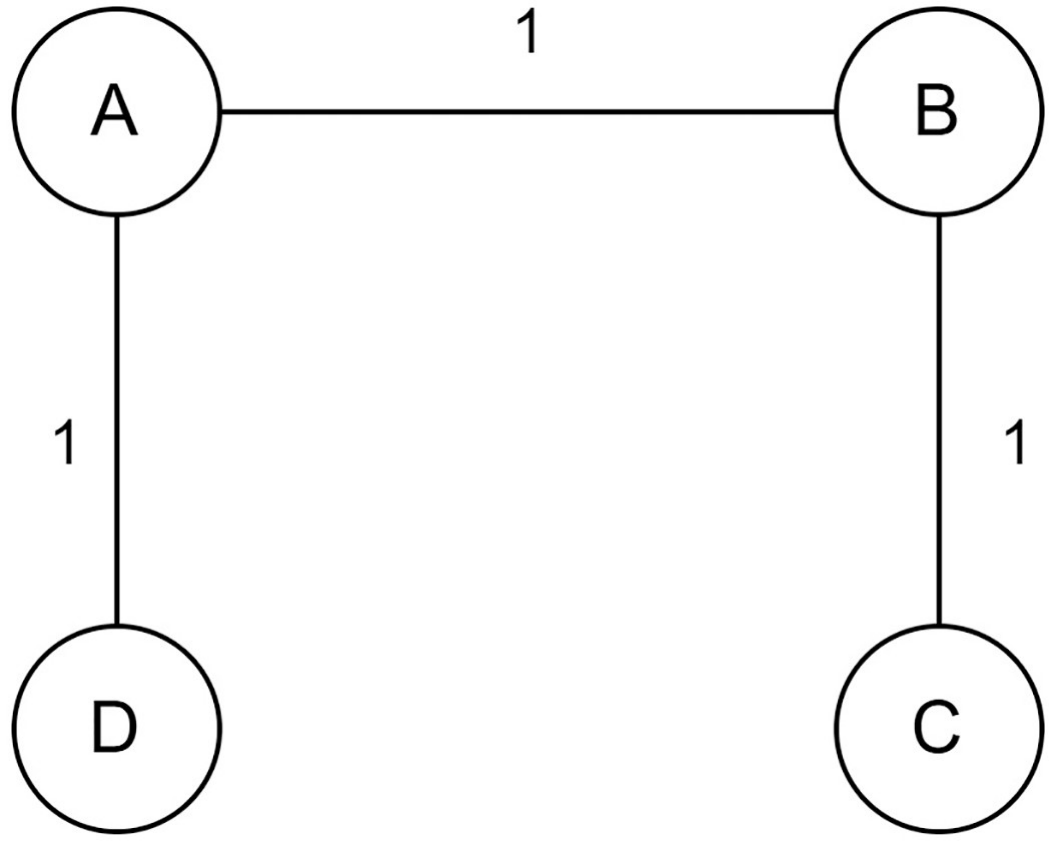
An example of the Kernighan-Lin algorithm.

Let’s take an example of a graph with 4 nodes. *V* = {*A*, *B*, *C*, *D*} as in Fig 3.

The Kernighan-Lin algorithm first divides the graph into two equal subsets {*A*, *B*} and {*C*, *D*}. The cut edge value for that is 2. The algorithm then swaps vertices between the two subsets to obtain a cut that has the lowest number of cut edges keeping the size of the subsets constant.

The algorithm checks the combinations {*A*, *C*}, {*B*, *D*} where the cut edge value is 3 and {*A*, *D*}, {*B*, *C*} where the cut edge value is 1. As, {*A*, *D*}, {*B*, *C*} is the partition with minimum cut, the algorithm returns this partition as the result. The algorithm returns {*A*, *D*}, {*B*, *C*} as output as it is the partition where two subsets have an equal number of vertices, and the cut is minimum. An example of the Kernighan-Lin algorithm is shown in Table 9.

**Table 9:** An example of the Kernighan-Lin algorithm.

Subsets	Cut edge value
Initial subset {{*A*, *B*}, {*C*, *D*}}	2
{{*A*, *C*}, {*B*, *D*}}	3
{{*A*, *D*}, {*B*, *C*}}	1

By changing the signs of all edge weights *c*_*ij*_, it is possible to transform the problem into maximizing the cost, e.g., obtaining maximum cut [48]. So, it is possible to obtain a local maximum cut by modifying the Kernighan-Lin partitioning algorithm. We have modified this algorithm to find the Max-cut by partitioning the graph. The pseudocode of this algorithm is shown in Algorithm 2.

**Algorithm 2**: Modified Kernighan-Lin algorithm.

1 **Input**: Graph, G(V, E)

2 **Output**: P(A, B)—partition of vertices that yields a local maximum cut

3 Randomly partition the vertices *V* into two sets *A* and *B*

4 Compute the initial cut value *T* of partition P(A, B)

5 **while** *gain* ≤ *0* **do**

6  compute costs *D* for all *a* ∈ *A* and *b* ∈ *B*

7  *gv*, *av*, *bv* ← ∅

8  **for** *n* ← 1 *to* |*V*|/2 **do**

9   find *a* ∈ *A* and *b* ∈ *B*, such that *gain* ← *D*[*a*] + *D*[*b*] − 2 × *c*(*a*, *b*) is maximal

10   remove *a* and *b* from further consideration in this pass

11   add *g* to *gv*, *a* to *av*, and *b* to *bv*

12   update costs *D* for the elements of *A* ← *A* − *a* and *B* ← *B* − *b*

13  **end**

14  find *k* which maximizes *gain*, the sum of *gv*[1], *gv*[2], …, *gv*[*k*]

15  **if** *gain* > 0 **then**

16   Exchange *av*[1], *av*[2], …, *av*[*k*] with *bv*[1], *bv*[2], …, *bv*[*k*]

17   *T* = *T* − *gain*

18  **end**

19 **end**

### Additional operator design

Studies have shown that the HHO algorithm may converge prematurely [49]. Therefore, adding additional operators may increase the HHO algorithm’s efficiency in the exploration phase [50]. To find out the better results we have to design several additional operators, these operators are described in the following sub-sections.

#### Refinement operator

Drawing inspiration from the decomposition operator used in the Chemical Reaction Optimization (CRO) algorithm [39], we have developed a new mechanism called the refinement operator. This adaptation aims to enhance the optimization process by improving the solution diversity and exploration capabilities of the Harris Hawks Optimization algorithm. The refinement operator prevents falling it into local optima.

**Fig 4:**
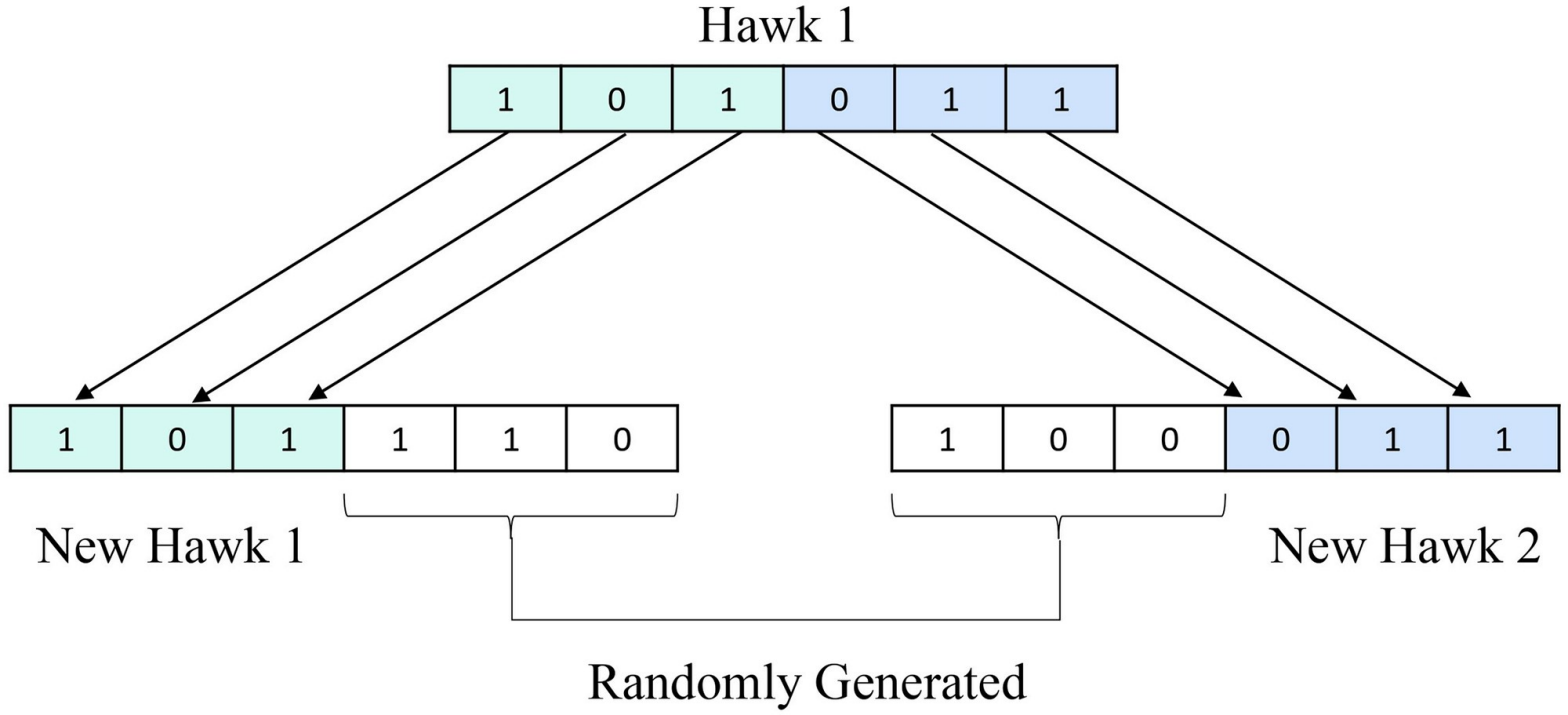
An example of the refinement operator.

In this operator, a single hawk gets split into two. Their structure is made more interesting with the addition of two newly produced hawks. Suppose hawk X produces hawks X1 and X2 as new offspring. The first portion of the hawk X generates a new hawk X1, while the remainder is chosen at random. The second part of the hawk X generates another hawk X2, and the remaining pieces are chosen at random. We are applying the refinement operator if a hawk does not update the result for 50 iterations. We have shown an example in Fig 4. The pseudocode of the refinement operator is provided in Algorithm 3.

**Algorithm 3**: Refinement operator.

1 **Input**: A Hawk

2 **Output**: A Better Hawk

3 *hawk*1, *hawk*2 ← *hawk*

4 **for** *i* ← 0 *to size*(*hawk*)/*2—1* **do**

5  *hawk*1[*i*] ← randomly 0 or 1

6 **end**

7 **for** *i* ← *size*(*hawk*)/*2 to size*(*hawk*)—*1* **do**

8  *hawk*2[*i*] ← random([0,])

9 **end**

10 **if** *objf*(*hawk*1) > *objf*(*hawk*2) **then**

11  **return** *hawk*1

12 **end**

13 **else**

14  **return** *hawk*2

15 **end**

#### Crossover operator

Liu et al. [51] introduced an enhanced Harris Hawk Optimization (HHO) algorithm known as CCNMHHO, which integrates the Nelder–Mead Simplex algorithm with a crisscross crossover technique, termed the horizontal and vertical crossover mechanism. The authors demonstrated the effectiveness of their approach in estimating parameters for photovoltaic systems.

**Fig 5:**
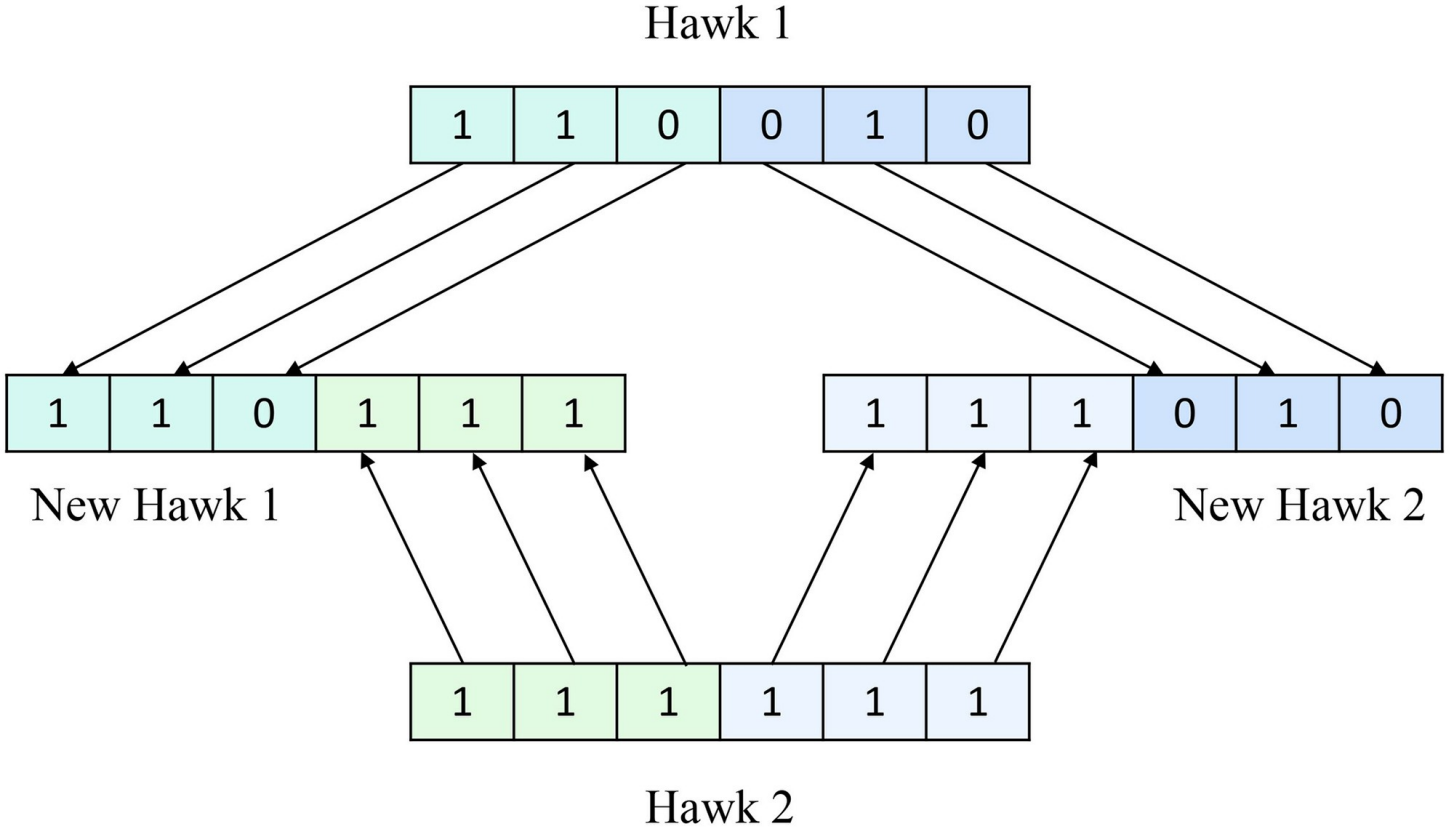
An example of a crossover operator.

The crossover operator consolidates two hawks to form a new hawk. We divided two hawks, *X*_1_ and *X*_2_, each into two halves. Then, we exchanged one half from *X*_1_ with one half from *X*_2_ to form two new hawks, *X*_*new*1_, and *X*_*new*2_. We apply the crossover operator if a hawk does not update the result for 50 iterations. Crossover enables the algorithm to explore new regions of the solution space by merging successful traits from both parents, potentially leading to better solutions. If one or both parents are high-quality solutions, the crossover operator can produce offspring that inherit beneficial characteristics. The pseudocode of the crossover operator is in Algorithm 4. Fig 5 demonstrates the example of the crossover operator.

**Algorithm 4**: Crossover operator.

1 **Input**: Population(*X*), two distinct hawks (*rand*1, *rand*2)

2 **Output**: Two modified hawks

3 *hawk*1 ← *X*[*rand*1], *hawk*2 ← *X*[*rand*2]

4 **for** *i* ← 0 *to size*(*hawk*1)/*2 -1* **do**

5  *hawk*1[*i*] ≔ *X*[*rand*2][*i*]

6 **end**

7 **for** *i* ← *size*(*hawk*2)/*2 to size*(*hawk*2)—*1* **do**

8  *hawk*2[*i*] = *X*[*rand*1][*i*]

9 **end**

10 **return** *hawk*1, *hawk*2

#### Mutation mechanism

Kardani et al. [52] presented an enhanced version of the Harris Hawks Optimization (HHO) algorithm integrated with Extreme Learning Machine (ELM), named ELM-IHHO. This innovative algorithm aims to address the shortcomings of traditional HHO by incorporating a mutation mechanism, thereby improving its performance in predictive tasks. Inspired by this, we also incorporate a mutation operator in our adaptation of the Harris Hawks Optimization (HHO) algorithm. This addition aims to enhance exploration capabilities and prevent premature convergence to local optima. We used the same mutation mechanism presented in [50] as follows:

$$\begin{array}{c} X_{new}=X_{rabbit}+F\left(\left(X_{r1}\left(t\right)-X_{r2}\left(t\right)\right)+\left(X_{r3}\left(t\right)-\left(X_{r3}\left(t\right)-X_{r4}\left(t\right)\right)\right)\right) \end{array}$$ (15)

*F* is the scaling factor in the equation, while *r*1, *r*2, *r*3, and *r*4 are distinct random numbers chosen from the range [1, N]. The number N reflects the total population. During the exploratory phase, the above equation is applied. Many models and algorithms employ mutation operators to increase optimization efficiency and global search capabilities [53–55], which inspired us to include this operator in the proposed technique. The description of the variables used in the Mutation phase is provided in Table 10.

**Table 10:** Description of variables used in mutation operation.

Variable	Description	Value
*X*_*new*_	New position of hawk	Generated from Eq 16
*X*_*rabbit*_	Location of the prey	Randomly generated
*F*	Scaling factor	Random value from 0 to 1
*r*1, *r*2, *r*3, *r*4	Random variables	Randomly generated within 1 to total population

#### Adjustment operator

The weights of the edges can contain specific values. An edge weight will be 0 if the edge is cut, otherwise, it will remain constant. In each iteration, we adjust the weights of the edges by clipping them inside the lower and upper ranges of our algorithm.

For example, if a hawk position contains negative values, clipping them inside the range will resolve the problem. Extremely high values will be resolved as well.

If the upper bound *ub* = 1 and lower bound *lb* = 0, for a matrix *M* = [−1.5, 0.5, −1.2, −0.07, 1.50.86, 0.92] then after adjustment the matrix will be *M* = [0, 0.5, 0, 0, 1, 0.86, 0.92]. The pseudocode of the operator is shown in Algorithm 5.

**Algorithm 5**: Adjustment operator.

1 **Input**: Hawk

2 **Output**: Modified hawk

3 **for** *i* ← *1 to size*(*hawk*) **do**

4  **if** *hawk*[*i*] < *lb* **then**

5   *hawk*[*i*] ← *lb*

6  **end**

7  **else if** *hawk*[*i*] > *ub* **then**

8   *hawk*[*i*] ← *ub*

9  **end**

10 **end**

#### Repair operator

We have used a repair operator for local search to enhance the performance of the HHO algorithm. We have maintained two arrays. The first array, *cut*[1…*V*], keeps track of the number of edges that have been cut where one endpoint is the vertex *i* in *cut*[*i*]. The other array, *uncut*[1…*V*] keeps track of the number of edges that not have been cut. Here, each array has a size of *V* where *V* is the total number of vertices.

**Fig 6:**
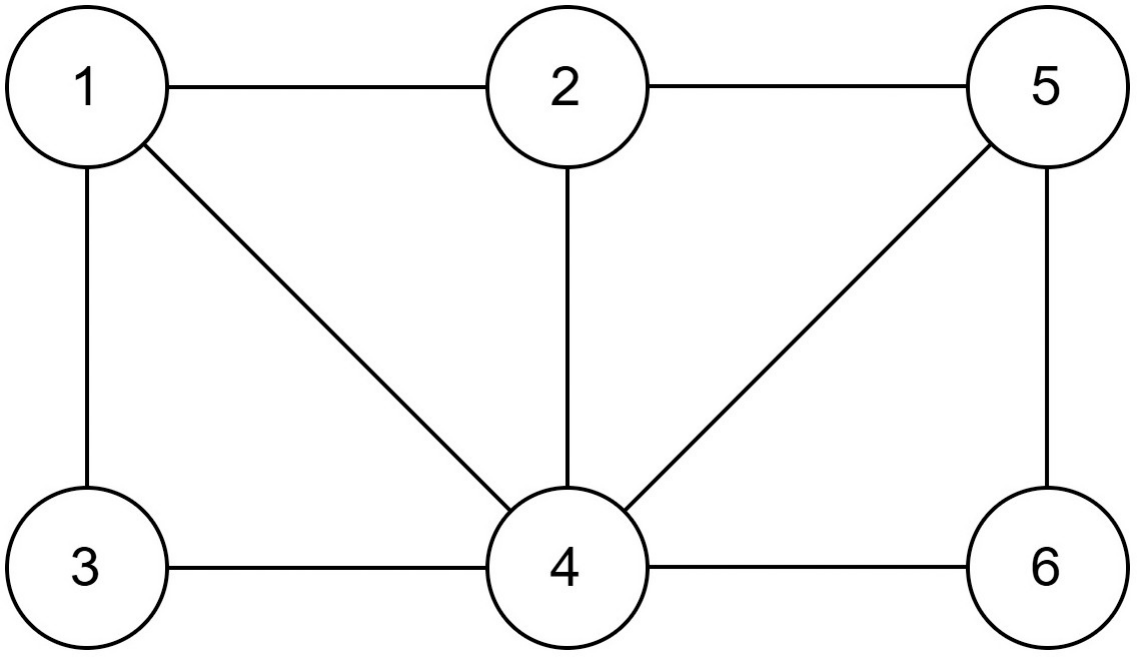
A random graph configuration.

**Fig 7:**
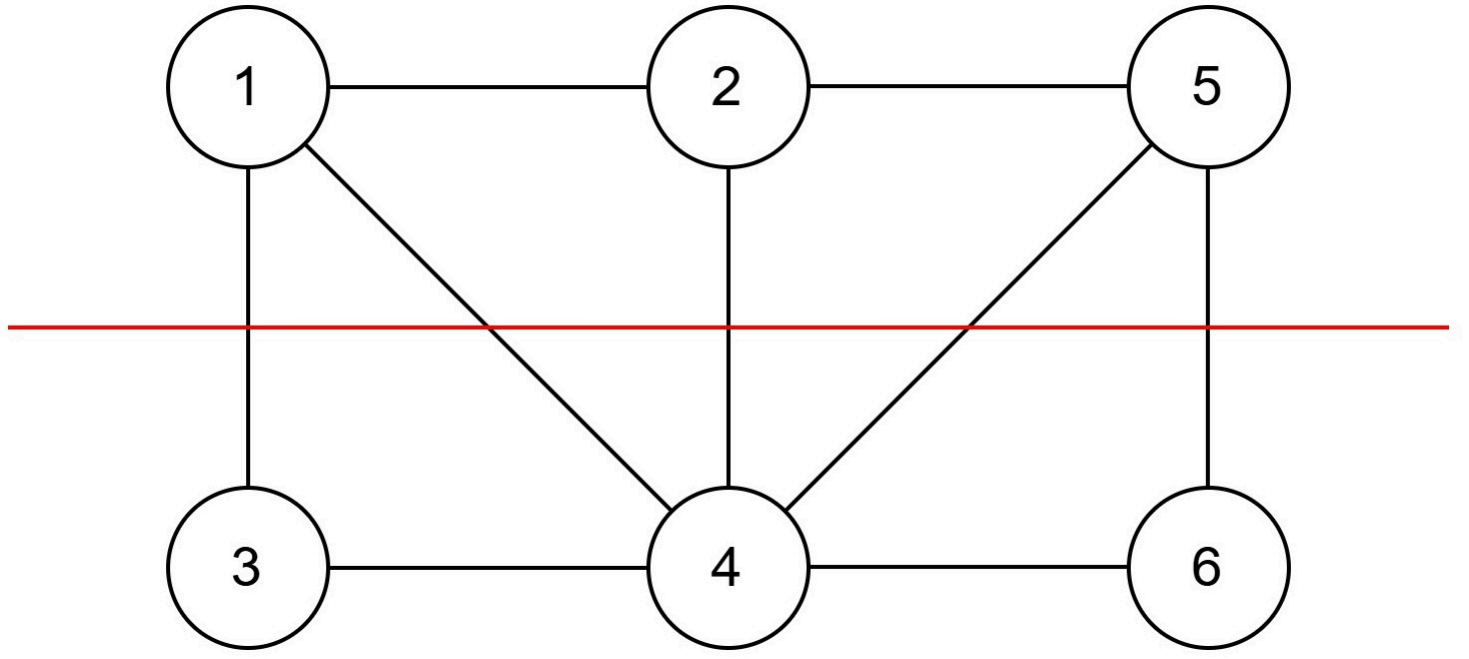
A random graph configuration with a cut.

For example, for the following graph in Fig 6, if a hawk configuration is [1, 1, 0, 0, 1, 0], from the cut graph in Fig 7, then the cut array = [2, 1, 1, 3, 2, 1], and uncut array = [1, 2, 1, 2, 1, 1].

In the repair operator, for each element in the hawk, a vertex has been moved from one subset to another (e.g., in a hawk, if the index of the hawk is 0, it is changed to 1, and vice-versa). The cut and uncut arrays have been modified accordingly. If the overall gain is positive, then we are accepting the change of the subset. Otherwise, the vertex remains in the subset in which it was before. We are calculating gain using the following formula:

Gain = Total changes in the sum of the elements of the cut array

**Fig 8:**
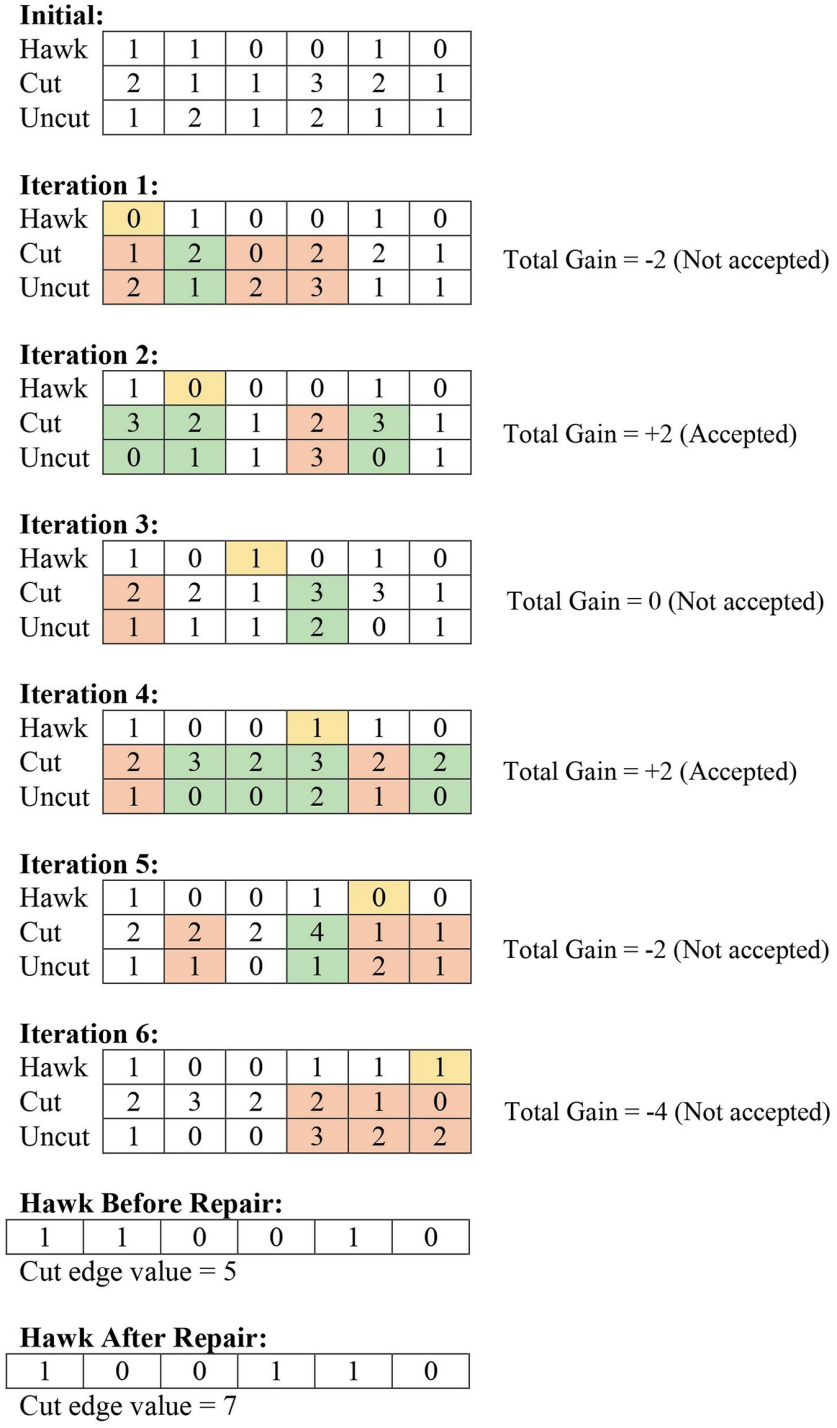
Working process of repair operator.

The pseudocode of the repair operator is shown in Algorithm 6, Algorithm 7, and Algorithm 8 for cut and uncut calculations. We have shown a pictorial view of the working process of the repair operator in Fig 8.

**Algorithm 6**: Repair operator.

1 Initialize totalArr = [1…*V*] with 0’s

2 **for** *edge* ← 0 *to size*(*edgeList*) **do**

3  *v*1 ← *edge*[0]

4  *v*2 ← *edge*[1]

5  Increment *totalArr*[*v*1] and *totalArr*[*v*2]

6 **end**

7 *cutArr* ← CalculateCut(*hawk*, *edgeList*)

8 *uncutArr* ← CalculateUncut(*totalArr*, *cutArr*)

9 **for** *I* ← 0 to *V* **do**

10  *temp* ← *hawk*

11 **end**

**Algorithm 7**: Cut calculation.

1 **Input**: hawk, edgeList

2 **Output**: cutArr

3 *cutArr* ← [1…*V*]

4 **for** *edge* ← 0 *to size*(*edgeList*) **do**

5  *v*1 ← *edge*[0]

6  *v*2 ← *edge*[1]

7 **end**

8 **if** *hawk*[*v*1] ≠ *hawk*[*v*2] **then**

9  Increment *cutArr*[*v*1] and *cutArr*[*v*2]

10 **end**

11 **return** *cutArr*

**Algorithm 8**: Uncut calculation.

1 **Input**: totalArr, cutArr

2 **Output**: uncutArr

3 *uncutArr* ← [1…*V*]

4 **for** *i* ← 0 *to* **do**

5  *V* *uncutArr*[*i*] ← *totalArr*[*i*] − *cutArr*[*i*]

6 **end**

7 **return** *uncutArr*

### Acceptance criterion

To ensure more effective optimization, we have introduced an acceptance criterion to obtain a better result. In each iteration, after operating through the algorithm, if the fitness value of a particular hawk improves, we accept that change. Otherwise, we simply keep the hawk as it was before the iteration. For example, if the fitness value of a hawk is 20 before an iteration and 18 after the iteration, the algorithm will keep the hawk position at 20.

### The overall algorithm

**Fig 9:**
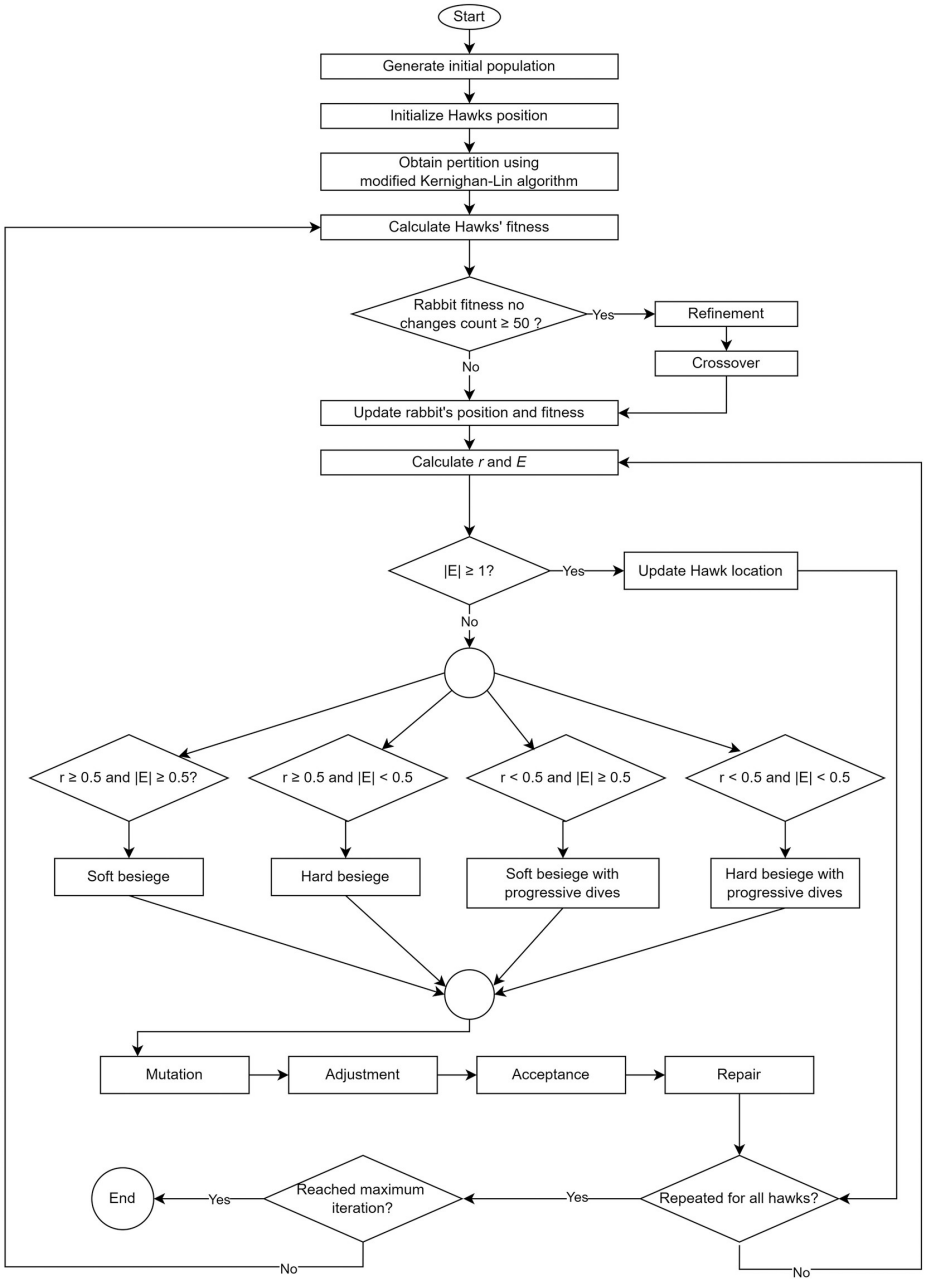
Flowchart of the proposed algorithm.

Unlike many other metaheuristic algorithms, HHO works on the initially generated population. After following the equations of HHO, it operates to find the optimal solution for the max-cut problem. The flowchart of the proposed method for the maximum cut problem using HHO is shown in Fig 9.

Pseudocode of the proposed algorithm is given in 9.

**Algorithm 9**: Max-Cut using Harris Hawks Optimization (MC-HHO) Algorithm

1 **Input**: Objective function *f*(*x*), Maximum number of iterations *MaxIter*, Population size *N*, Graph *G*(*V*, *E*), Upper and lower bounds *lb*, *ub*

2 **Initialize** population *X*_*i*_ (*i* = 1, 2, …, *N*) with random positions within bounds [*lb*, *ub*]

3 Obtain initial partition using modified Kernighan-Lin algorithm

4 Evaluate fitness of each hawk *X*_*i*_ using *f*(*x*)

5 Find the current best solution *X*_best_

6 **for** *t* = 1 *to MaxIter* **do**

7  **if** *PreyNoUpdateCount* ≥ *50* **then**

8   *X*_*i*_ = refinement(*X*_*i*_)

9   *X*_*i*_, *X*_*rand*_ = crossover(*X*_*i*_, *X*_*rand*_)

10  Update prey energy, $E=2\times (1-\frac{t}{MaxIter})$

11  **for** *each hawk* *X*_*i*_ **do**

12   **if** |*E*| ≥ 1 **then**

13    Generate random values *q* and *r*

14    Update *X*_*i*_ using Eq 2

15   **else**

16    **if** *r* ≥ 0.5 *and* |*E*| ≥ 0.5 **then**

17     Soft besiege: Update *X*_*i*_ using Eq 5

18    **else if** *r* ≥ 0.5 *and* |*E*| < 0.5 **then**

19     Hard besiege: Update *X*_*i*_ using Eq 7

20    **else if** *r* < 0.5 *and* |*E*| ≥ 0.5 **then**

21     Soft besiege with progressive dives: Update *X*_*i*_ using Eq 12

22    **else**

23     Hard besiege with progressive dives: Update *X*_*i*_ using Eq 13

24   *X*_*i*_ = mutation(*X*_*i*_)

25   adjustment(*X*_*i*_, *lb*, *ub*)

26   Evaluate updated fitness of *X*_*i*_

27   Check acceptance criteria of *X*_*i*_

28   *X*_*i*_ = repair(*X*_*i*_)

29  Update the best solution *X*_best_ if needed

30 **Output**: Best solution *X*_best_ and its fitness

## Experimental results and discussion

In this section, we have shown the results produced by our proposed approach and the comparison with the other related state-of-the-art methods.

### Dataset collection

To test and solve the max-cut problem, G-set datasets have been used in the proposed method as graph data. Helmberg and Rendl built the G-set dataset using a graph generator [56]. We have chosen 33 G-set instances. The graphs in these datasets are described by information on the edges and vertices. The first row reflects the size of the graph that is the total number of vertices *V* and edges *E*. The first value is the size of the vertices and the second one is the number of edges in the first row. The third column of the dataset represents the weight of the edges. We converted the graph to a 1-D array to work with it.

### Experimental environment

We implemented our proposed algorithm (MC-HHO) using Python 3.12 on a system equipped with an Intel Core i7-7700 CPU @ 3.60 GHz, 24 GB of RAM, and a Linux environment. The hardware supports parallel multithreading processing, enhancing computational efficiency for large-scale problems. Some cases require a large amount of memory, so we used the Linux PC running Ubuntu 22.04 LTS, which offers greater virtual memory management.

### Parameters initialization

Four parameters of HHO are initialized to start the process. The value of the maximum number of iterations, upper bound, lower bound, and total number of hawks in the population are the commencing values of the proposed method that are shown in Table 11.

**Table 11:** Optimal values of parameters in the proposed method.

Parameters	Description	Values
*T*	Maximum number of iterations	3000
*N*	Number of Hawks (Search Agent)	100
*Lb*	Lower Bound	0
*Ub*	Upper Bound	1

### Parameter tuning

There are two types of tuning used here. One is iteration and another is search agent tuning.

#### Iteration tuning

**Fig 10:**
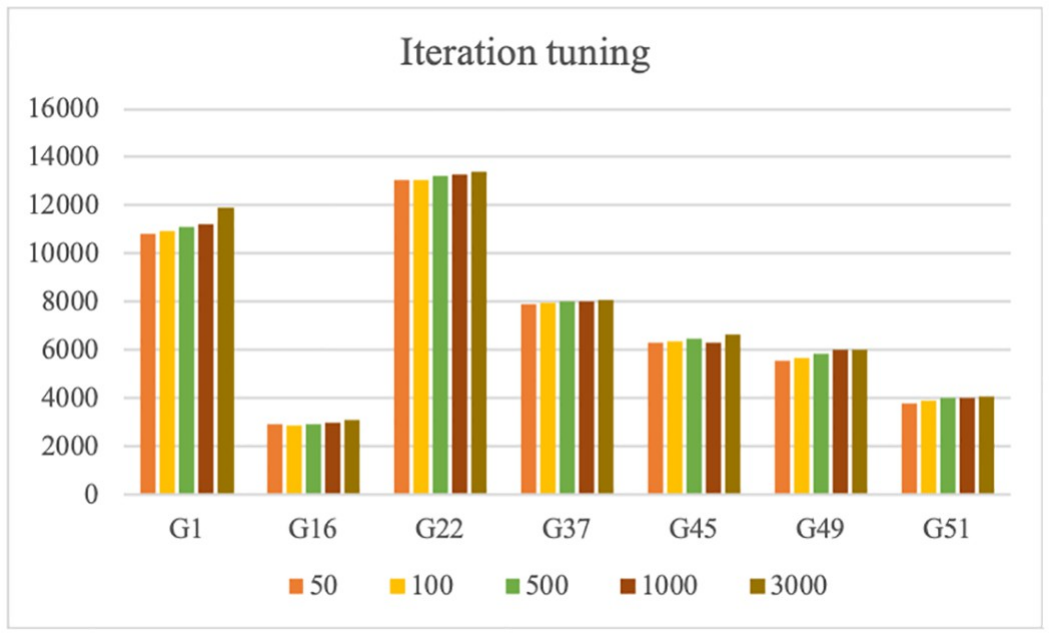
Iteration tuning using different values.

For tuning the number of iterations, we tested 50, 100, 500, 1000, and 3000 iterations. In some instances, optimal results were achieved within fewer iterations, such as 100 or 1000, while in others, it required 2500 or 2600 iterations to reach the best outcome. However, the performance plateaued for 2500 or more iterations. Therefore, 3000 is selected as the maximum number of iterations to obtain the best result for all instances. Fig 10 shows the tuning of iteration using five different values for seven different instances.

#### Search agent tuning

**Fig 11:**
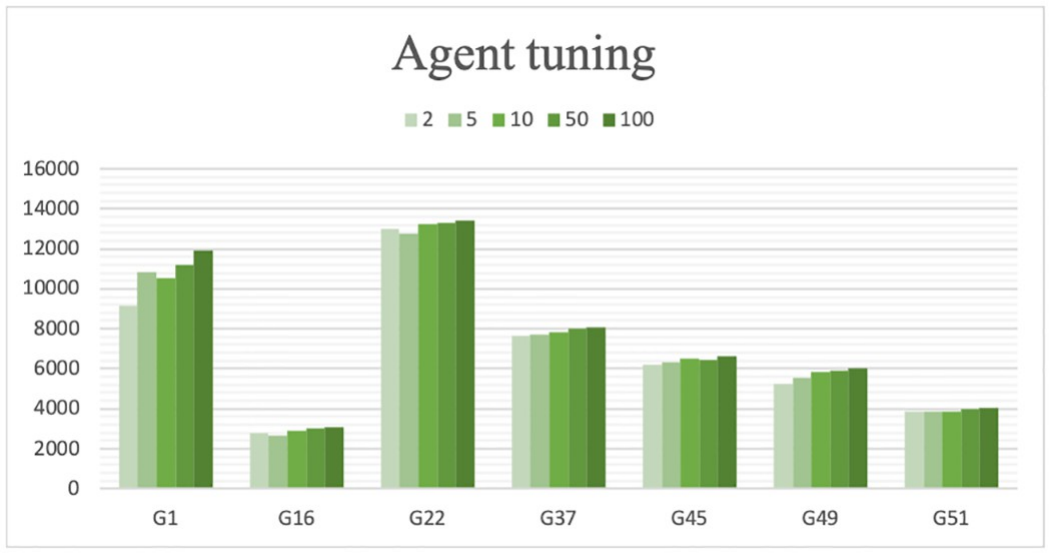
Search agent tuning using different values.

To determine the optimal number of search agents, we tested each instance with the values of *SearchAgentNo*: 2, 5, 10, 50, and 100. The results indicated that increasing the *SearchAgentNo* improved the performance, but the improvement became very small compared to the increase in execution time. As the number of hawks increases, the algorithm takes longer to reach a solution. Thus *SearchAgentNo* is taken as 100 empirically. Fig 11 shows the tuning of search agents using five different values for seven different instances.

### Experimental results comparison

In this section we have compared our obtained results with the results acquired by the Discrete Cuckoo Search with Local Search for Max-cut Problem (DCSLS) [57], PSO-EDA [17], and TSHEA [15] algorithms. According to Tables 12 and 13, it is clear that HHO is successfully obtaining the maximum cut values that are near the best-known values in most cases.

**Table 12:** Comparison of results with DCSLS, MC-HHO, and MC-HHO without additional operator.

Instances	Nodes	Edges	DCSLS (a)	MC-HHO (b)	MC-HHO (c) (without operators)	Gap(b-a)	Gap(b-c)
G1	800	19176	11607	11912	11502	305	410
G2	800	19176	11599	11620	11504	21	116
G3	800	19176	11605	11622	11534	17	88
G11	800	1600	530	564	544	34	20
G12	800	1600	528	556	512	28	44
G13	800	1600	552	582	578	30	4
G14	800	4694	3035	3064	3012	29	52
G15	800	4661	3016	3050	2977	34	73
G16	800	4672	3018	3053	3000	35	53

**Table 13:** Comparison of the results of the TSHEA, PSO-EDA, and MC-HHO.

Instances	Nodes	Edges	TSHEA(a)	PSO-EDA(b)	MC-HHO(c)	Gap(c-a)	Gap(c-b)
G1	800	19176	11624	11624	11912	288	288
G2	800	19176	11620	11620	11620	0	0
G3	800	19176	11622	11622	11622	0	0
G4	800	19176	11646	11646	11718	72	72
G5	800	19176	11631	11631	11680	49	49
G11	800	1600	564	564	564	0	0
G12	800	1600	556	556	556	0	0
G13	800	1600	582	582	582	0	0
G14	800	4694	3064	3062	3064	0	2
G15	800	4661	3050	3050	3050	0	0
G16	800	4672	3052	3052	3053	1	1
G17	800	4667	3047	3047	3047	0	0
G22	2000	19990	13359	13359	13393	34	34
G23	2000	19990	13344	13344	13344	0	0
G24	2000	19990	13337	13337	13337	0	0
G25	2000	19990	13340	13338	13340	0	2
G26	2000	19990	13328	13326	13303	-25	-23
G35	2000	11778	7687	7685	7687	0	2
G36	2000	11766	7680	7671	7680	0	9
G37	2000	11785	7691	7678	8063	372	385
G38	2000	11779	7688	7688	7670	-18	-18
G43	1000	9990	6660	6660	6660	0	0
G44	1000	9990	6668	6650	6668	18	18
G45	1000	9990	6654	6654	6654	0	0
G46	1000	9990	6649	6649	6649	0	0
G47	1000	9990	6661	6657	6661	4	4
G48	3000	6000	6000	6000	6000	0	0
G49	3000	6000	6000	6000	6000	0	0
G50	3000	6000	5880	5880	5880	0	0
G51	1000	5909	4031	3848	4031	183	183
G52	1000	5916	3841	3851	3841	-10	-10
G53	1000	5914	3838	3850	3838	-12	-12
G54	1000	5916	3852	3850	3852	0	2

We have also shown the gaps between the results. The gap is the difference between the best result obtained by MC-HHO and the best result obtained by the compared method. Mathematically,

$$\begin{array}{c} \text{Gap}\;=\;\text{number}\;\text{of}\;\text{cuts}\;\text{from}\;\text{MC-HHO}\;-\;\text{number}\;\text{of}\;\text{cuts}\;\text{from}\;\text{compared}\;\text{method} \end{array}$$ (16)

Table 12 shows that the number of cuts in MC-HHO is higher than DCSLS in all instances. Additionally, we compared the performance of MC-HHO without the refinement and crossover operators, labeled as “MC-HHO without Operator (c)” in the table. The results indicate that the proposed MC-HHO, which incorporates both operators, achieves a slight but consistent improvement in the quality of cuts across all instances compared to the version without these operators. This improvement demonstrates the impact of the refinement and crossover operators in enhancing the exploration and exploitation balance of the algorithm.

Table 13 demonstrates the results for TSHEA, PSO-EDA, and MC-HHO. It shows the number of cuts in each instance and the gaps between TSHEA and MC-HHO, as well as PSO-EDA and MC-HHO. A greater number of cuts signifies improved performance. According to Eq 16, a positive gap value indicates superior performance for MC-HHO, while a negative value means lower performance. If the gap is zero, it means that the performance of MC-HHO is equal to that of the compared algorithms.

Table 14 presents the gap comparison of DCSLS, TSHEA, and PSO-EDA against MC-HHO. From the table, the superiority over DCSLS is clear as there is no negative gap as well as there is a positive gap for every instance. This means for every instance MC-HHO performs better than DCSLS. The results indicate that the MC-HHO generates a greater number of cuts than the DCSLS across nine instances, with a total difference of 533 cuts. In comparison with TSHEA and MC-HHO, we see that for most of the instances, MC-HHO performs better. In nine instances MC-HHO obtained positive gaps that are 1021 more cuts than the TSHEA in total. However, in four instances, the performance of MC-HHO is lower than the TSHEA. MC-HHO gets 65 lower cuts (i.e. negative gaps) than TSHEA. Overall, MC-HHO prevailed by obtaining 956 cuts more than TSHEA. In the same four instances as the TSHEA algorithm, MC-HHO obtained lower cuts than the PSO-EDA algorithm. It achieved 1051 more cuts in 14 instances than the PSO-EDA in total. This performance helps the MC-HHO to attain the overall 988 cuts more than the PSO-EDA algorithm.

**Table 14:** Summary of comparison of the results of the DCSLS, TSHEA, PSO-EDA, and MC-HHO.

Method	Aspects	# Instances	Total gaps
DCSLS vs MC-HHO	Positive gaps	9	533
	Equal results	0	0
	Negative gaps	0	0
	Overall enhanced	9	533
TSHEA vs MC-HHO	Positive gaps	9	1021
	Equal results	20	0
	Negative gaps	4	65
	Overall enhanced	33	956
PSO-EDA vs MC-HHO	Positive gaps	14	1051
	Equal results	15	0
	Negative gaps	4	63
	Overall enhanced	33	988

### Comparison of time with PSO-EDA

We have implemented the proposed method (MC-HHO) and the method PSO-EDA in the same machine. The comparison is shown in Table 15.

**Table 15:** Comparison of the time for the PSO-EDA and MC-HHO on G-set dataset.

Instances	PSO-EDA (sec)	MC-HHO (sec)	Difference
G1	35.714	7.803	27.911
G2	34.367	7.920	26.447
G3	34.441	8.070	26.371
G4	30.228	7.979	22.249
G5	31.339	7.537	23.802
G11	3.048	2.478	0.570
G12	3.348	2.704	0.644
G13	3.023	2.393	0.630
G14	7.274	3.395	3.879
G15	6.969	3.877	3.092
G16	6.369	3.500	2.869
G17	6.479	3.781	2.698
G22	49.566	22.162	27.404
G23	44.828	22.172	22.656
G24	46.959	24.602	22.357
G25	43.246	23.848	19.398
G26	42.254	22.592	19.662
G35	27.680	16.303	11.377
G36	27.582	14.753	72.829
G37	27.474	13.742	13.732
G38	27.154	14.482	12.672
G43	13.438	5.479	7.959
G44	13.548	5.426	8.122
G45	13.062	5.335	7.727
G46	12.125	5.196	6.929
G47	13.165	5.569	7.596
G48	24.098	31.642	-7.544
G49	12.386	45.362	-32.976
G50	12.647	32.395	-19.748
G51	6.125	4.266	1.859
G52	6.155	4.471	1.684
G53	6.113	4.214	1.899
G54	6.782	4.399	2.383

**Fig 12:**
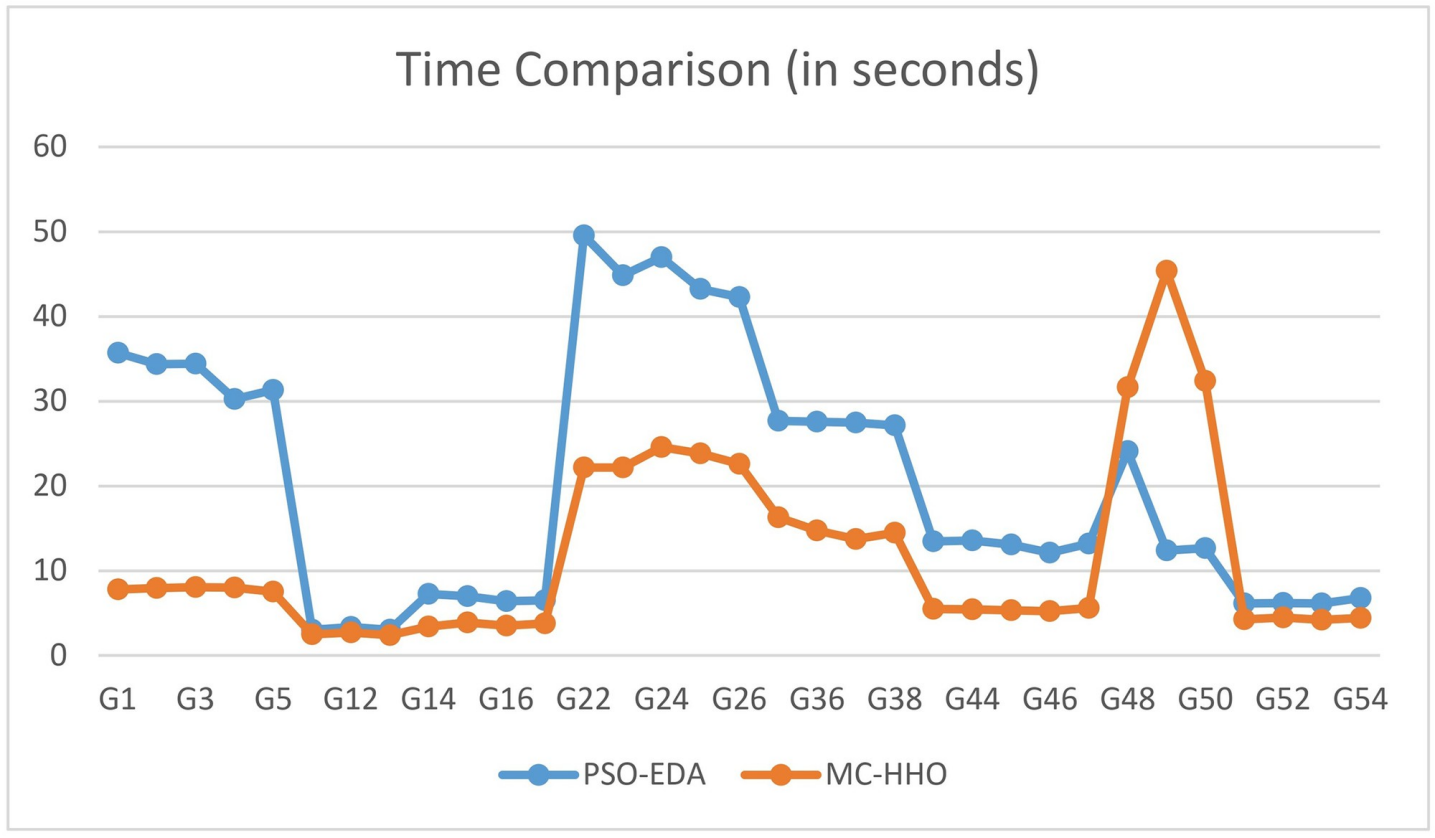
Time comparison between PSO-EDA and MC-HHO.

The running times of our proposed MC-HHO are less than that of PSO-EDA in almost all the cases except for two instances—G48 to G50. We have shown the time comparison between PSO-EDA and our proposed algorithm in Fig 12.

### Time complexity analysis

The time complexity of the HHO algorithm can generally be broken down into three components—a) Initialization: This typically requires O(n) time, where *n* is the number of hawks in the population. b) Fitness Evaluation: For each iteration, the fitness of each hawk is evaluated. The Fitness function can be varied in different implementations. If fitness function takes O(f) times, then for *m* iterations, the total time will be O(m.n.f). c) Updating Positions: this usually takes O(n) for each iteration. Therefore, the overall time complexity of the HHO algorithm is O(m.n.f).

The proposed system can be divided into three parts. The first part is initialization in which the elementary population has been introduced, the hawk position is initialized, the Kernighan-Lin graph partitioning algorithm is used to partition the graph and the hawk fitness is calculated. In Algorithm 1 the first iteration occurs based on the total number of vertices. If the total number of vertices is *v* then the time complexity will be O(v). For creating graph G of V vertices, the time complexity is $O(v^{2})$. For the Kernighan-Lin graph partitioning algorithm, the time complexity is $O(v^{2}\;\text{log}\;v)$. Every other operator has single loops and runs sequentially. As there are no nested loops in any other algorithms the maximum taken time from the operators is O(v). Thus the maximum time taken for the initialization part is $O(v^{2}\;\text{log}\;v)$.

The second part is iterations and fitness evaluation. Based on the value of maximum iteration and search agent number the time complexity varies. If the number of search agents is *m* and the maximum iteration is *n* then the time taken by the proposed algorithm is $O(m\times n\times v+m\times v^{2}\;\text{log}\;v)$.

Lastly, in the updating part, there are no nested loops. Therefore, the time complexity of updating *n* hawks is O(n). So the upper boundary of the time is $O(mnv+mv^{2}\;\text{log}\;v)$. Simplifying to the dominant term (omitting lower-order terms), the time complexity can be expressed as $O(mv^{2}\;\text{log}\;v)$.

### Statistical analysis

**Fig 13:**
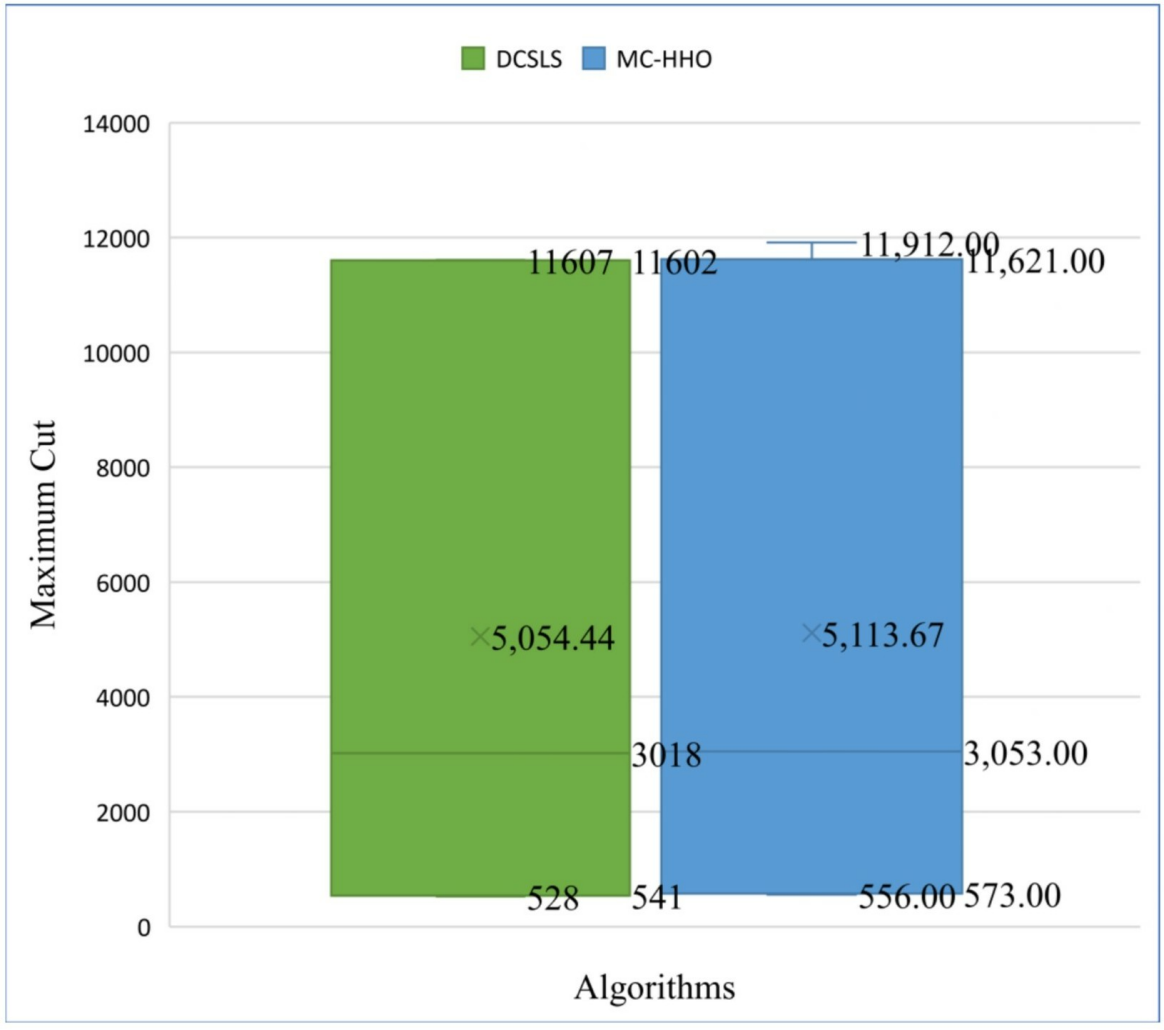
Box plot comparison between DCSLS and MC-HHO.

The result of the proposed approach is compared using statistical analysis with other algorithms. To show the performance of the current research statistically box plot has been used. Fig 13 shows the box plot comparison of DCSLS and MC-HHO. According to the box plot the maximum, minimum, median, and average values obtained from the MC-HHO are greater than DCSLS suggesting superior performance. Moreover, the mean value for MC-HHO is 5113.67 whereas it is 5054.44 for DCSLS, indicating that MC-HHO consistently achieves better results on average.

The values of the box plots are shown in Table 16.

**Table 16:** Box plot values of DCSLS and MC-HHO.

	DCSLS	MC-HHO
**Min**	528	556
**First quartile (Q1)**	541	573
**Median**	3018	3053
**Third quartile (Q3)**	11602	11621
**Max**	11607	11912
**Mean**	5054.44	5113.67

**Fig 14:**
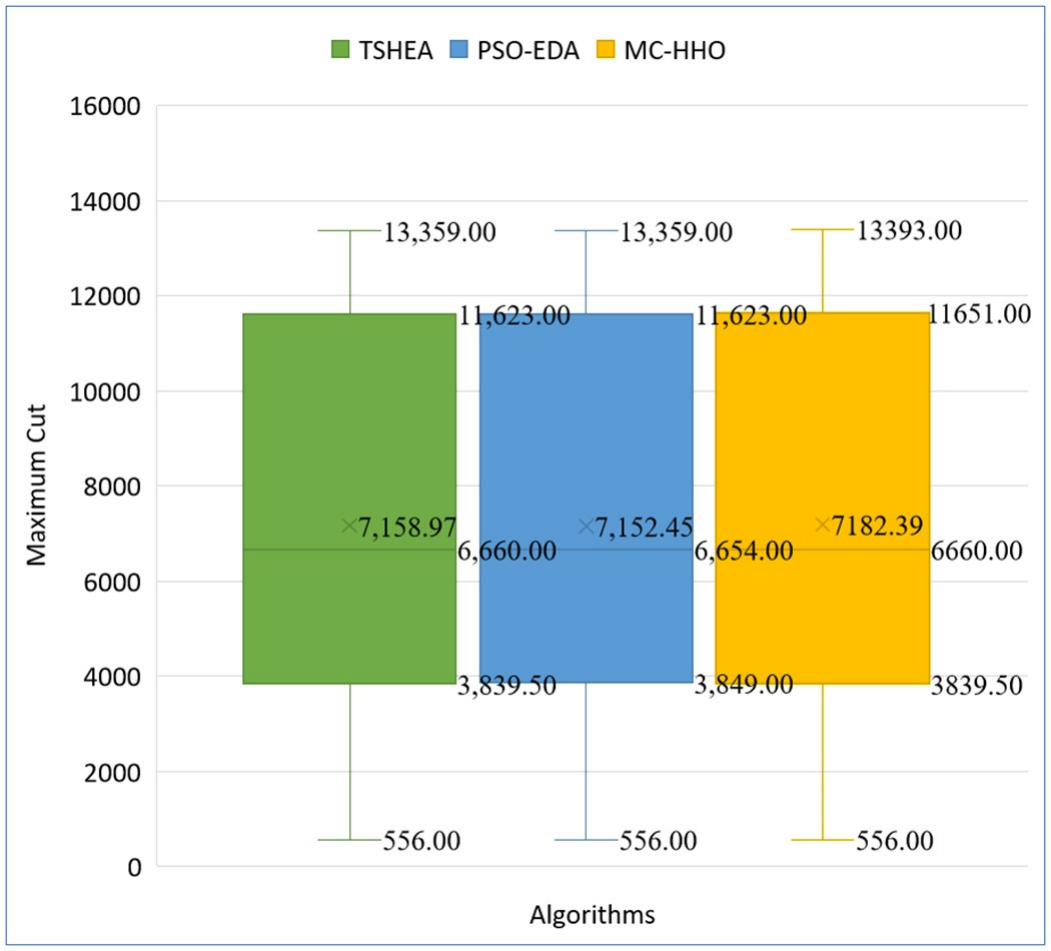
Box plot comparison between TSHEA, MC-HHO, and PSO-EDA.

Similarly, Fig 14 shows the box plot comparison of TSHEA, MC-HHO, and PSO-EDA. According to the box plot the maximum, median, and average values obtained from the MC-HHO are greater than both TSHEA and PSO-EDA. The mean value for MC-HHO is 7182.39, surpassing TSHEA and PSO-EDA’s means, at 7158.97 and 7152.45, respectively. This indicates that MC-HHO consistently outperforms the other two algorithms on average. The higher mean indicates that MC-HHO produces a more reliable solution for maximizing the cut value, making it a more robust method for solving the problem compared to TSHEA and PSO-EDA.

The values of the box plots are shown in Table 17.

**Table 17:** Box plot values of TSHEA, MC-HHO, and PSO-EDA.

	TSHEA	MC-HHO	PSO-EDA
**Min**	556	556	556
**First quartile (Q1)**	3839.5	3839.5	3849
**Median**	6660	6660	6654
**Third quartile (Q3)**	11623	11651	11623
**Max**	13359	13393	13359
**Mean**	7158.97	7182.39	7152.45

### Non-parametric statistical significance test

To test the significance of the outcome on the G-set dataset of the proposed method statistically, the Wilcoxon signed-rank test is applied to the MC-HHO and TSHEA, and MC-HHO and PSO-EDA pairs. The Friedman test is applied to the MC-HHO, TSHEA, and PSO-EDA.

#### Wilcoxon signed-rank test

According to the Wilcoxon signed-rank test, the proposed approach performs significantly better than the two other methods. The significance level for the test is set at 0.05, which is used to compare two related samples and assess the ranks of their population means, commonly referred to as a two-tailed hypothesis.

Table 18 provides the statistical significance of the proposed method using the Wilcoxon signed-rank test for the G-set dataset compared to TSHEA and PSO-EDA respectively.

**Table 18:** Wilcoxon signed-rank test for G-set dataset.

	MC-HHO and TSHEA	MC-HHO and PSO-EDA
**W-value**	19.5	39.5
**Mean Difference**	-11264.69	-8135.61
**Sum of positive ranks**	71.5	131.5
**Sum of negative ranks**	19.5	39.5
**Z-value**	-1.817	-2.0033
**Mean (W)**	45.5	85.5
**Standard Deviation (W)**	14.31	22.96
**Sample Size (N)**	13	18
**p-value**	.06876	.0455
**Result**	Significant	Significant

According to Table 18, for MC-HHO and TSHEA pair sample size is 13 and the p-value is 0.06876. For the p-value less than 0.05, the result becomes insignificant. However as the p-value is greater than 0.05, the result is significant for the sample size 13. Besides, for the pair MC-HHO and PSO-EDA, the sample size is 18, and for the p-value 0.0455, the result is significant.

#### Friedman test

The Friedman test is applied to the results using MC-HHO, TSHEA, and PSO-EDA of the G-set dataset. According to the test, the result is insignificant as the *p*–*value* is less than the significance level that is 0.05. Table 19 refers to the ranks of every comparable algorithm based on the value of the max-cut obtained as the output of the algorithms. According to the total rank of every algorithm, it is clear that the proposed technique outperforms every other method. The *p*–*value* from the Friedman test measured from the ranks of the algorithms is 0.14056. Thus the result is not significant at *p* < 0.05. From the test, the *χ*^2^ value is also calculated as 3.9242.

**Table 19:** Ranks of the algorithms for Friedman test of G-set dataset.

Ranks (MC-HHO)	Ranks (TSHEA)	Ranks (PSO-EDA)
3	1.5	1.5
2	2	2
2	2	2
3	1.5	1.5
3	1.5	1.5
2	2	2
2	2	2
2	2	2
2.5	2.5	1
2	2	2
3	1.5	1.5
2	2	2
3	1.5	1.5
2	2	2
2	2	2
2.5	2.5	1
1	3	2
2.5	2.5	1
2.5	2.5	1
3	2	1
1	2.5	2.5
2	2	2
3	1.5	1.5
2	2	2
2	2	2
3	1.5	1.5
2	2	2
2	2	2
2	2	2
3	1.5	1.5
1	2.5	2.5
1	2.5	2.5
2.5	2.5	1
Sum: 73.5	Sum: 67	Sum: 57.5

## Conclusions and future works

Combinatorial optimization-based problems are very hard to solve using exact algorithms especially when the number of instances is large. Thus to solve these types of problems metaheuristic algorithms can be used. As the max-cut problem is one of the combinatorial optimization-based problems, we have proposed a nature-based metaheuristic algorithm called Harris Hawk Optimization (HHO) to solve it. The main challenge to solve this problem is to design the three HHO phases. To obtain a better outcome, hybridization with refinement and crossover operators has been combined with HHO. An adjustment operator has been used to improve the results. We have also used a mutation mechanism and acceptance criteria to obtain better performance. Our proposed method gives better results in most cases. For many instances of the graphs, the proposed method obtains the best-known results. It is tested for both small and large scales of instances of datasets. From the experimental results, it is observed that the proposed approach outperforms any other known related existing methods for solving the max-cut problem both in terms of results and time. The statistical significance test has also been provided to prove the superiority of the proposed system. The results of this research have important real-world implications for addressing combinatorial optimization problems, particularly in areas such as network design, VLSI, and machine learning. Although the outcome using the proposed hybrid method is better than previous research works, it could find the optimal solution for every instance on the G-set dataset. Besides, there is scope to find the outcome for other datasets like MQLIB. Future work should focus on enhancing the algorithm’s scalability and exploring new datasets to improve performance further.
